# Data Augmentation Enhances Plant-Genomic-Enabled Predictions

**DOI:** 10.3390/genes15030286

**Published:** 2024-02-24

**Authors:** Osval A. Montesinos-López, Mario Alberto Solis-Camacho, Leonardo Crespo-Herrera, Carolina Saint Pierre, Gloria Isabel Huerta Prado, Sofia Ramos-Pulido, Khalid Al-Nowibet, Roberto Fritsche-Neto, Guillermo Gerard, Abelardo Montesinos-López, José Crossa

**Affiliations:** 1 Facultad de Telemática, Universidad de Colima, Colima 28040, Colima, Mexico; osval78t@gmail.com (O.A.M.-L.); msolis2@ucol.mx (M.A.S.-C.); 2 International Maize and Wheat Improvement Center (CIMMYT), Km 45, Carretera Mexico-Veracruz, Texcoco 52640, Edo. de México, Mexico; l.crespo@cgiar.org (L.C.-H.); c.saintpierre@cgiar.org (C.S.P.); g.gerard@cgiar.org (G.G.); 3 Independent Researcher, Zinacatepec 75960, Puebla, Mexico; glory_chav@hotmail.com; 4 Centro Universitario de Ciencias Exactas e Ingenierías (CUCEI), Universidad de Guadalajara, Guadalajara 44430, Jalisco, Mexico; sofdgo@gmail.com; 5 Distinguish Scientist Fellowship Program and Department of Statistics and Operations Research, King Saud University, Riyah 11451, Saudi Arabia; knowibet@ksu.edu.sa; 6 Louisiana State University, Baton Rouge, LA 70803, USA; rfneto@agcenter.lsu.edu; 7 Colegio de Postgraduados, Montecillo 56230, Edo. de México, Mexico

**Keywords:** genomic selection, plant breeding, data augmentation, novel approach

## Abstract

Genomic selection (GS) is revolutionizing plant breeding. However, its practical implementation is still challenging, since there are many factors that affect its accuracy. For this reason, this research explores data augmentation with the goal of improving its accuracy. Deep neural networks with data augmentation (DA) generate synthetic data from the original training set to increase the training set and to improve the prediction performance of any statistical or machine learning algorithm. There is much empirical evidence of their success in many computer vision applications. Due to this, DA was explored in the context of GS using 14 real datasets. We found empirical evidence that DA is a powerful tool to improve the prediction accuracy, since we improved the prediction accuracy of the top lines in the 14 datasets under study. On average, across datasets and traits, the gain in prediction performance of the DA approach regarding the Conventional method in the top 20% of lines in the testing set was 108.4% in terms of the NRMSE and 107.4% in terms of the MAAPE, but a worse performance was observed on the whole testing set. We encourage more empirical evaluations to support our findings.

## 1. Introduction

Meeting the demands of the expanding global population is an imperative undertaking that requires a substantial increase in food production. For this reason, plant breeding is essential to ensure food, economic, and environmental sustainability, as well as to contribute to human health and well-being. Nevertheless, achieving this production increase is a multifaceted endeavor, impeded by the depletion of natural resources, limited arable land availability, and the considerable variability in climate conditions, among other challenges. Consequently, innovative solutions, exemplified by the genomic selection (GS) methodology introduced by Meuwissen [1], have become essential for genetic enhancements. These advancements are geared towards bolstering the stability of yields, elevating productivity, increasing resistance to diseases, and enhancing nutritional profiles and ultimate end-use quality across pivotal crops such as wheat, rice, maize, and various others [2].

Genomic selection (GS) and prediction (GP) represent a groundbreaking paradigm shift in the realm of plant breeding [3]. Nevertheless, the practical execution of GS remains a formidable task, as it does not consistently ensure highly accurate predictions [4]. High prediction accuracies are essential for the successful implementation of genomic prediction in plant breeding for several crucial reasons: (1) efficient selection. Accurate predictions enable breeders to efficiently identify and select individuals with desired traits, such as a high yield, disease resistance, or nutritional quality. This streamlines the breeding process by reducing the need to grow and evaluate large numbers of plants. (2) Resource optimization. High-accuracy predictions help allocate limited resources such as land, labor, and financial investments more effectively. Breeders can focus their efforts on plants with the greatest potential for improvement, thus conserving resources. (3) Faster progress. When predictions are highly accurate, progress in breeding programs is accelerated. This allows for the development of improved crop varieties in a shorter timeframe, which is crucial to address food security and agricultural challenges [5]. (4) Cost reduction. Accurate predictions reduce the costs associated with field trials, extensive phenotyping, and maintaining large breeding populations. This cost reduction can make breeding programs more economically viable. (5) Genetic gain. Higher prediction accuracies lead to greater genetic gains, meaning that the desirable traits are more rapidly and effectively incorporated into the breeding population [5], which results in crops with improved characteristics. (6) Stability. Stable and reliable predictions minimize the risk of selecting plants with undesirable traits, which could set back breeding programs or lead to inferior varieties [5]. (7) Confidence. High-accuracy predictions provide breeders with confidence in their selections, increasing the likelihood of success and the adoption of new varieties by farmers [6].

Reaching a high prediction accuracy with GS is challenging due to genetic complexity, environmental variations, and limitations in data and resources. Complex traits often involve multiple genes, while environmental factors impact trait expression [7,8,9,10,11]. Accurate phenotyping and marker data are crucial, and overfitting and the population structure can hinder accuracy. Ongoing research aims to improve models, marker densities, and data quality to enhance the precision of genomic predictions [7,8,9,10,11]. For this reason, novel approaches are required to improve the prediction accuracy of GS.

In general, deep neural networks are trained to minimize error on the training dataset (minimizing practical risk), and these neural networks scale linearly with the size of the training set [12]. However, learning theory says that the error in the training dataset is minimized when the size of the training set does not increase. On the other hand, Zhang [12] pointed out that training sets used to minimize the error changed the outcome when evaluated in practical examples with trained sets outside the standard dimensional range. In practical terms, the conclusion is that minimizing the error on the training set alone does not explain the cases when the training data differ slightly from the testing distribution.

Zhang [12] argued that for different sizes of training sets, the choice for training the training set is data augmentation (DA) that is based on the vicinal (neighborhood) minimization risk principle for each training set. Then, the virtual training sets can be obtained from the vicinity distribution of the training set. That is, for the optimization of the training set in GS, a vicinity distribution of the training set is required. However, Zhang [12] pointed out that, although DA improves generalization, the method is highly data-dependent and does not model the vicinity relationships across different possible training populations. Based on this, Zhang [8] presented a DA routine named *mixup* that constructs different training set examples. Essentially, we need to find a function that describes the relationship of a random feature vector (X) and a random target vector (Y) to define a joint probability distribution P(X,Y). *mixup* trains a neural network that regularizes the neural networks that favor simple linear regressions between diverse training sets.

Data augmentation (DA) is a novel technique that artificially increases the training set to improve the prediction performance. The training set is artificially expanded by applying various transformations on the existing data, such as rotations, flips, or cropping. This enhances model robustness, generalization, and performance by exposing it to a wider range of training examples, thus improving its ability to handle real-world variations and noise [13,14,15,16]. Some successful applications of data augmentation are (1) image classification. DA is widely used in image classification tasks, such as recognizing objects or animals, by creating variations of images with different angles, lighting, and perspectives [17]. (2) Natural language processing (NLP). In NLP, DA techniques such as synonym replacement, paraphrasing, and text generation are applied to expand text datasets, improving the performance of models in tasks such as sentiment analysis and text summarization [18]. (3) Speech recognition. DA is used in speech recognition by altering audio samples with noise, speed variation, or pitch shifts, making models more robust to different speaking styles and environments [19]. (4) In the context of tabular data, DA involves generating synthetic data points by slightly perturbing or interpolating existing data entries [13]. For example, in financial fraud detection, one can augment a dataset of credit card transactions by creating new instances with slightly modified transaction amounts or timestamps to help train a model to detect fraudulent activities more effectively.

Regarding the average gain in prediction performance, using DA compared to not using has different effects, depending on the specific dataset, task, and augmentation techniques used. In general, DA can lead to notable improvements in prediction performance, particularly when the original dataset is limited or when the task involves recognizing patterns in noisy or diverse data. The degree of improvement can range from a few percentage points to substantial enhancements, making DA a valuable tool to enhance the robustness and generalization of machine learning models. However, the extent of the gain will depend on factors such as the quality of the augmentation techniques, the complexity of the task, and the size and quality of the initial dataset [20].

Due to these factors, plant breeding can greatly benefit from the strategic use of DA to enhance the accuracy of genomic prediction models. By augmenting limited datasets with variations and synthetic examples, breeders can inject diversity and representation into their training data. This approach can allow models to capture a broader spectrum of genetic and phenotypic traits, leading to more robust and accurate predictions of plant performance. Furthermore, DA is of paramount importance to mitigate overfitting and reduces the risk of models learning from rare, biased, or unrepresentative samples [13,14,15,16]. In the context of genomic selection, where the availability of large-scale genomic data is often limited, DA can be a valuable tool to maximize the utility of existing data. It can empower breeders to make more informed decisions, accelerate the breeding cycle, and ultimately contribute to the development of improved plant varieties with higher yields, better disease resistance, and enhanced adaptability to changing environmental conditions.

The significance of delving into DA within the context of plant breeding cannot be overstated. This research endeavor aims to harness the potential of data augmentation to elevate predictive performance, crucial to the successful adoption of genomic selection (GS) methodologies. The practical implementation of GS remains a formidable challenge, as it does not always guarantee consistent high-quality predictions. Data augmentation techniques present an effective remedy, offering a pragmatic means to bolster the prediction accuracy. By generating synthetic data points that expand the training dataset, DA introduces vital diversity and enriches the representation of genetic variations. In an era where genomics plays an increasingly pivotal role in modern agriculture and breeding endeavors, embracing DA holds the promise of uncovering novel insights, expediting breeding cycles, and fueling advancements in crop improvement. Ultimately, this pursuit significantly contributes to the overarching goals of global food security and sustainable agricultural practices.

## 2. Materials and Methods

### 2.1. Datasets

We used fourteen datasets to evaluate the methods proposed in this study. A summary of the fourteen datasets is provided in Table 1.

### 2.2. GBLUP Model

To determine the prediction accuracy for the traits of interest, the popular Bayesian GBLUP model [6] was implemented with the following predictor:

(1) $$Y_{i}=\mu +g_{i}+\epsilon_{i}\;\;\;\;\;\;\;\;\;\;\;\;\;\;$$

where $Y_{i}$ represents the continuous response variable measured in the ith line, μ is a general intercept, $g_{i}$ denotes the random effects of genotypes distributed exactly as $g={\left(g_{1},\ldots ,g_{J}\right)}^{T}∼N_{J}\left(0,\sigma_{g}^{2}G\right)$, where G is the genomic relationship matrix (linear kernel), computed as proposed by Vanraden [21], and $\epsilon_{i}$ are random error components in the model assumed to be independent normal random variables with a mean of 0 and variance of $\sigma_{e}^{2}$. The implementation of this model was carried out in R statistical software (R-4.3.2) [22] using the BGLR library of Pérez and de los Campos [23].

### 2.3. Data Augmentation Method

There are many data augmentation techniques, but we will focus on the *mixup* method, introduced in the paper titled “*mixup*: Beyond Empirical Risk Minimization” by Zhang et al. [12] that offers a domain-agnostic approach to enhancing machine learning models. Under this method, synthetic data are generated using the following mathematical formulas:

(2) $$\tilde{x}=\lambda x_{i}+(1-\lambda {)x}_{j}\;$$

where $x_{i}$ and $x_{j}$ are rows (vectors) of lines i and j with its corresponding marker information; each vector has a length of p.

(3) $$\tilde{y}=\lambda y_{i}+(1-\lambda {)y}_{j}$$

where $y_{i}$ and $y_{j}$ are scalars of the phenotypic (BLUEs) response variable of lines i and j. $(x_{i}$, $y_{i}$) and ($x_{j}$, $y_{j}$) represent two randomly selected lines from our training dataset, with λ ∈ [0, 1] denoting a mixing coefficient. In this research, we used λ=0.5. In essence, *mixup* enriches the training distribution by integrating the inherent understanding that linear interpolations of feature vectors should correspond to linear interpolations of their corresponding target values. Notably, the implementation of *mixup* is highly efficient, requiring only a few lines of code and incurring minimal computational overhead. It is important to point out that in our approach, the synthetic data were generated only from the top 20% of the lines in the training set and the models with augmented data were trained using only the top 20% of the lines in the training set + the resulting synthetic data generated from the top 20% of the top lines in the training as input. When the GBLUP model given in Equation (1) was trained using the augmented data, the results are denoted as “A = Augmented”, whereas when the original training set was used for training, we denote the results as “C = Conventional”. The G required in the GBLUP model (Equation (1)) was computed with the augmented inputs for the Augmented approach and using the original markets (inputs) under the Conventional approach.

### 2.4. Evaluation of Prediction Performance

The evaluation methodology used in this research entailed a cross-validation technique known as “random-partition-line”. In this cross-validation technique, during each fold, the data from 20% of the lines were designated as the test set, while the data from all other lines collectively formed the training set (80%), as elucidated by Montesinos-López et al. [6]. The number of folds was 10 and the average of the 10 folds was reported as the prediction performance.

Two metrics were used to evaluate the genomic ability of the models. One metric is the normalized root mean square error (NRMSE) and the other one is the mean arctangent absolute prediction error (MAAPE). The mean square error is calculated by MSE = 1T(∑i=1T(yi−f^(xi))2), where $y_{i}$ denotes the ith observed value, while f^(xi) represents the ith predicted value for observation. The normalized root mean square error was calculated by $NRMSE=\frac{RMSE}{\bar{y}}$, where RMSE=1T(∑i=1T(yi−f^(xi))2) was used as a metric to evaluate the prediction accuracy.

The MAAPE provides a measure of the prediction accuracy by considering the arctangent of the absolute errors between the predicted and actual values. It is useful when evaluating the performance of predictive models, especially in situations where dealing with percentage errors might be problematic. MAAPE = 1n∑i=1narctanyi − f^(xi)yi, where $\;y_{i\;}$ and f^(xi) represent the observed and predicted values of the *i*th cultivar, respectively.

## 3. Results

### 3.1. Organizing Data and Results

**Figure 1:**
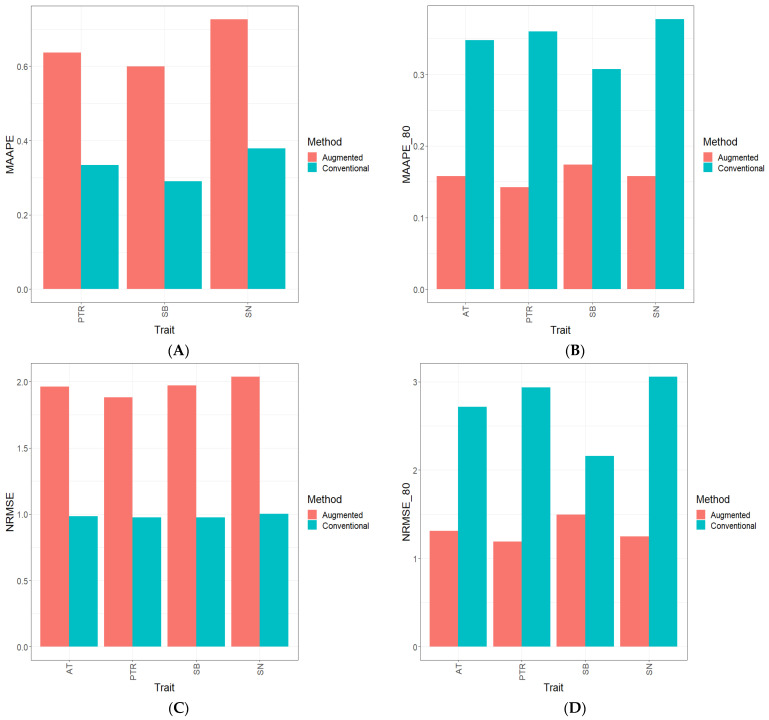
Prediction performance results for Dataset 1 (**Disease**) using the Conventional (green) and Augmented (red) methods in terms of (**A**) the mean arctangent absolute percentage error (MAAPE) for all the testing set, (**B**) the mean arctangent absolute percentage error for the top 20% (MAAPE_80, quantile 80%) of the testing set, (**C**) the normalized root mean square error (NRMSE) for the entire testing set, and (**D**) the normalized root mean square error for the top 20% of the testing set (NRMSE_80 quantile 80%).

**Figure 2:**
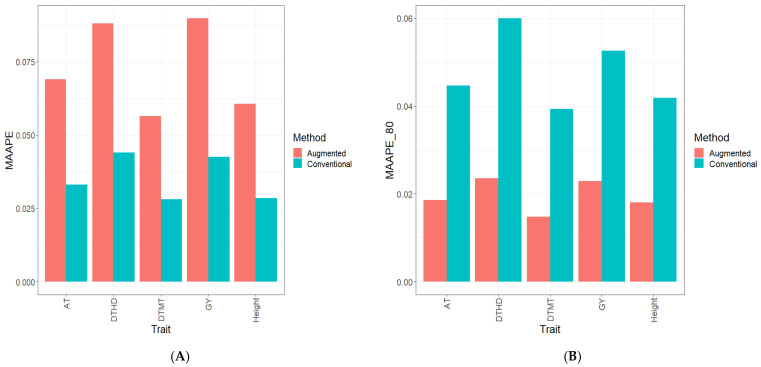

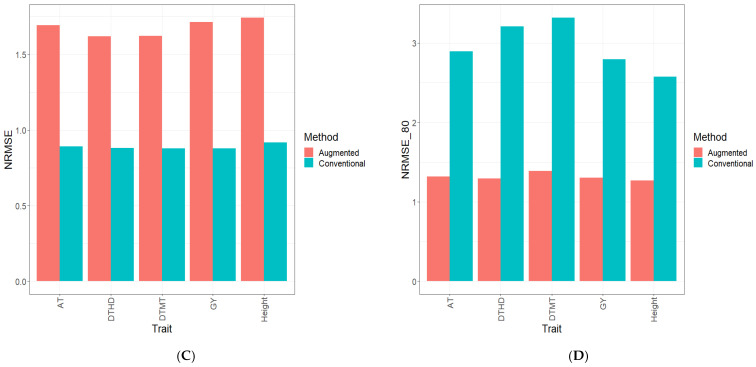
Prediction performance results for Dataset 2 (**EYT_1**) using the Conventional (green) and Augmented (red) methods in terms of (**A**) the mean arctangent absolute percentage error (MAAPE) for all the testing set, (**B**) the mean arctangent absolute percentage error for the top 20% (MAAPE_80, quantile 80%) of the testing set, (**C**) the normalized root mean square error (NRMSE) for the entire testing set, and (**D**) the normalized root mean square error for the top 20% of the testing set (NRMSE_80 quantile 80%).

**Figure 3:**
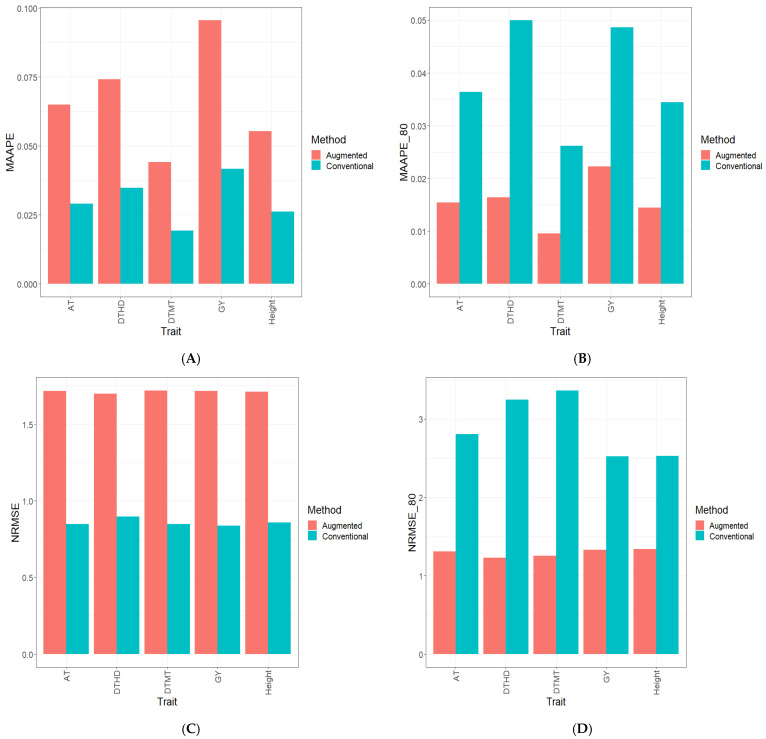
Prediction performance results for Dataset 3 (**EYT_2**) using the Conventional (green) and Augmented (red) methods in terms of (**A**) the mean arctangent absolute percentage error (MAAPE) for all the testing set, (**B**) the mean arctangent absolute percentage error for the top 20% (MAAPE_80, quantile 80%) of the testing set, (**C**) the normalized root mean square error (NRMSE) for the entire testing set, and (**D**) the normalized root mean square error for the top 20% of the testing set (NRMSE_80 quantile 80%).

**Figure 4:**
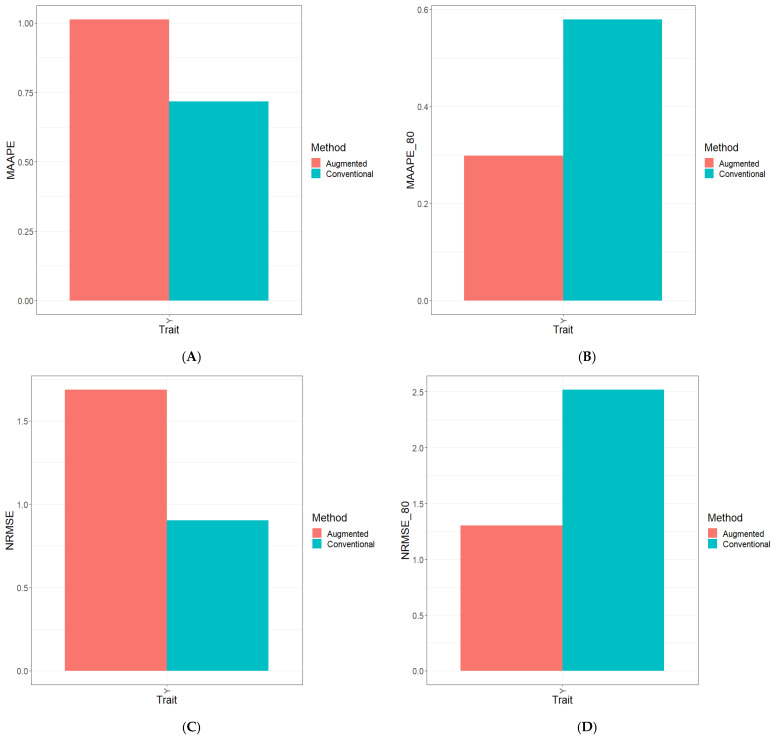
Prediction performance results for Dataset 5 (**Maize**) using the Conventional (green) and Augmented (red) methods in terms of (**A**) the mean arctangent absolute percentage error (MAAPE) for all the testing set, (**B**) the mean arctangent absolute percentage error for the top 20% (MAAPE_80, quantile 80%) of the testing set, (**C**) the normalized root mean square error (NRMSE) for the entire testing set, and (**D**) the normalized root mean square error for the top 20% of the testing set (NRMSE_80 quantile 80%).

**Figure 5:**
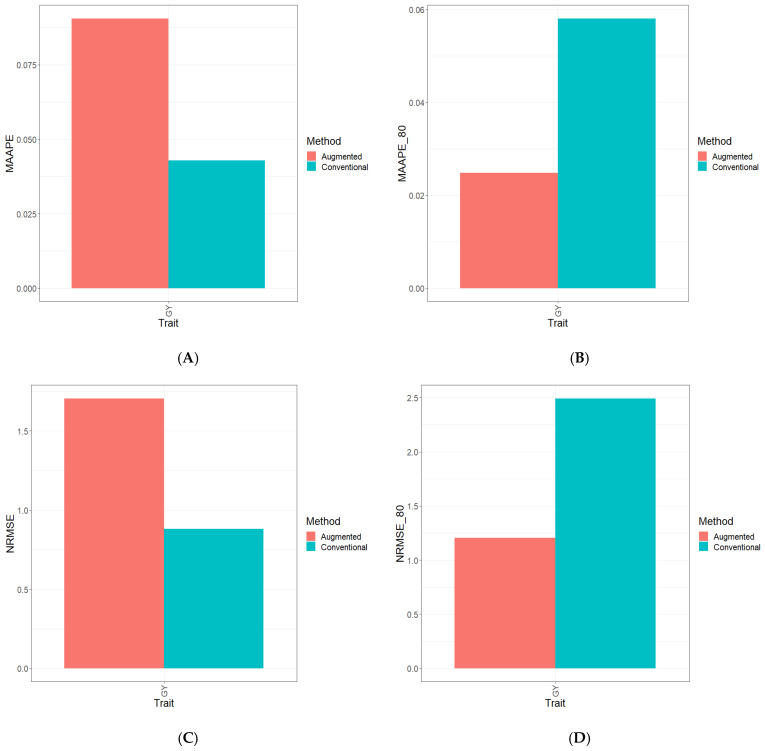
Prediction performance results for Dataset 6 (**Wheat_1**) using the Conventional (green) and Augmented (red) methods in terms of (**A**) the mean arctangent absolute percentage error (MAAPE) for all the testing set, (**B**) the mean arctangent absolute percentage error for the top 20% (MAAPE_80, quantile 80%) of the testing set, (**C**) the normalized root mean square error (NRMSE) for the entire testing set, and (**D**) the normalized root mean square error for the top 20% of the testing set (NRMSE_80 quantile 80%).

**Figure 6:**
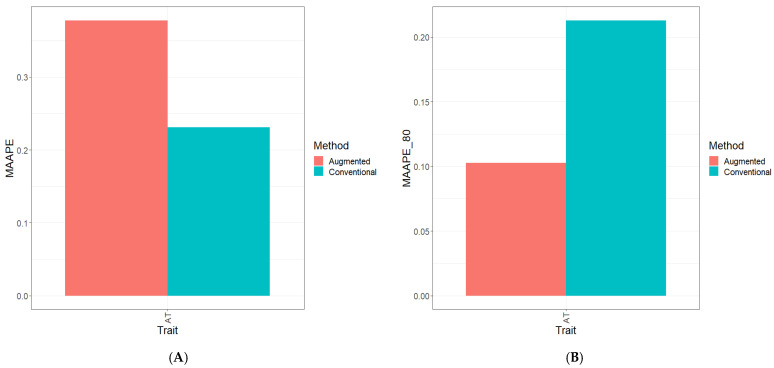

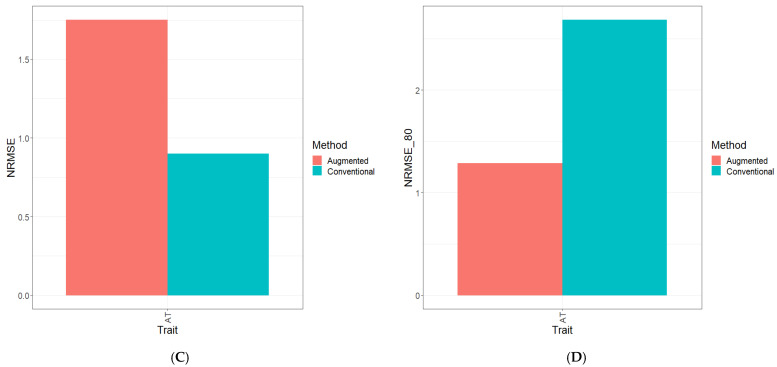
Prediction performance results for Across_Data dataset using the Conventional (green) and Augmented (red) methods in terms of (**A**) the mean arctangent absolute percentage error (MAAPE) for all the testing set, (**B**) the mean arctangent absolute percentage error for the top 20% (MAAPE_80, quantile 80%) of the testing set, (**C**) the normalized root mean square error (NRMSE) for the entire testing set, and (**D**) the normalized root mean square error for the top 20% of the testing set (NRMSE_80 quantile 80%).

Part of the results are provided in six sections. Results Section 3.2, Section 3.3, Section 3.4, Section 3.5 and Section 3.6 describe the results for Dataset 1 (Disease), Dataset 2 (ETY_1), Dataset 3 (EYT_2), Dataset 5 (Maize), and Dataset 6 (Wheat_1). Section 3.7 contains a summary of all datasets. Figure 1, Figure 2, Figure 3, Figure 4, Figure 5 and Figure 6 contain four sub-figures (A, B, C, and D), displaying different genomic prediction performances. (**A**) shows the mean arctangent absolute percentage error (MAAPE) for all the testing sets, (**B**) shows the mean arctangent absolute percentage error for the top 20% (MAAPE_80, quantile 80%) of the testing set, (**C**) shows the normalized root mean square error (NRMSE) for the entire testing set, and (**D**) shows the normalized root mean square error (NRMSE) for the top 20% of the testing set (NRMSE_80 quantile 80%).

Furthermore, results for Dataset 1 (Disease), Dataset 2 (ETY_1), Dataset 3 (EYT_2), Dataset 5 (Maize), Dataset 6 (Wheat_1), and all datasets are also given in the Supplementary Material, which contains results from all the other nine datasets (Dataset 4 and Datasets 7–14 (Wheat_2–6, Indica, Japonica, and groundnut)). The Supplementary Materials contain figures with the observed and predicted cultivars using the Conventional and DA methods for (**A**) the plots generated for the entire testing set using the Conventional method, (**B**) the plots generated for the top 20% of the testing set using the Conventional method, (**C**) the plots generated for the total testing set using the Augmented method, and (**D**) the plots generated for the top 20% of the testing set using the Augmented method. For example, for Dataset 1 (Disease), the Supplementary Material contains Figures S1–S3 for traits PTR, SB, and SN, respectively, and Table S1, showing the traits, the method, and the values of the NRMSE, MAAPE, NRMSE_80%, and MAAPE_80 (quantile 80%, prediction of 20% as the testing set). The Supplementary Material contains the results across all sites, see Table Across data at the Supplementary Material. Furthermore, the results for the rest of the nine datasets (Dataset 4 (EYT_3) and Datasets 7–14 (EYT_3, Wheat 2–6, Indica, Japonica, and Groundnut, respectively)) are shown only in the Supplementary Material.

### 3.2. Dataset 1 Disease

Figure 1A and Figure 1C show that when the two metrics (MAAPE and NRMSE) were computed using the complete testing set, the prediction performance for each trait (PTR, SB, and SN) and across traits (AT) was better under the Conventional method. In terms of the NRMSE and MAAPE in the PTR trait, the C method was better than the A method by 48.1% and 47.5%, respectively. Meanwhile, in trait SB, the C method was better than the A method by 50.6% (NRMSE) and 51.7% (MAAPE), respectively. Also, in trait SN, the C method outperformed the A method by 50.8% (NRMSE) and 47.9% (MAAPE), respectively. Finally, across traits (ATs), we can also observe that the C method was superior to the A method by 49.9% (NRMSE) and 48.9% (MAAPE). (See details in Table S1 in the Supplementary Materials.)

In Figure 1B and Figure 1D, an examination of the computed metrics, NRMSE and MAAPE, reveals a noteworthy trend. These metrics were exclusively derived from the top 20% of the testing dataset. It becomes evident that, in every evaluated trait (PTR, SB, and SN), as well as across all traits (ATs), the A method exhibited a substantial performance advantage over the C method. In the PTR trait, the A method surpassed the C method by a substantial margin, with improvements of 146.6% in the NRMSE and 152.8% in the MAAPE. Turning to the SB trait, the A method displayed superiority with a 44.1% reduction in the NRMSE and a 76.4% decrease in the MAAPE compared to the C method. Furthermore, in the SN trait, the A method displayed a remarkable performance improvement, outperforming the C method by 145.2% in the NRMSE and 139.1% in the MAAPE. Extending across traits (ATs), the A method remained the superior choice, boasting a 107.1% reduction in the NRMSE and a 120.2% decrease in the MAAPE when compared to the C method (See details in Table S1 of Supplementary Materials). Also, Figures S1–S3 in the Supplementary Materials display the behavior of the observed and predicted values under both methods (C and A) for three traits (PTR, SB, and SN, respectively).

### 3.3. Dataset 2 EYT_1 Data

Figure 2A and Figure 2C illustrate the outcomes of computing two metrics, namely the MAAPE and NRMSE, using the entire testing dataset. The Conventional method outperformed the novel methods both for individual traits (DHTD, DTMT, GY, and height) and across all traits (ATs). Specifically, in the DHTD trait, the C method presented superior results compared to the A method, with improvements of 45.6% in the NRMSE and 50.0% in the MAAPE. Similarly, for the DTMT trait, the C method surpassed the A method by 45.8% in the NRMSE and 50.0% in the MAAPE. The trend continued in the GY and height traits, where the C method outperformed the A method by 48.8% and 47.4% in the NRMSE, and 52.8% and 53.3% in the MAAPE, respectively. Across all traits (ATs), the superiority of the C method was evident, with a 47.3% improvement in the NRMSE and a 51.5% improvement in the MAAPE. See details in Table S2 (Supplementary Materials).

Moving on to Figure 2B and Figure 2D, a distinct pattern emerges when considering the NRMSE and MAAPE metrics, which were exclusively calculated from the top 20% of the testing dataset. Interestingly, the A method consistently outperformed the C method in all evaluated traits (DHTD, DTMT, GY, and height), as well as across all traits (ATs). Notably, in the DHTD trait, the A method showcased substantial improvements over the C method, with reductions of 148.0% in the NRMSE and 156.5% in the MAAPE. Likewise, for the DTMT trait, the A method displayed superiority, with a 140.4% decrease in the NRMSE and a remarkable 178.6% reduction in the MAAPE compared to the C method. This pattern persisted in the GY trait, where the A method’s performance lead was prominent, exceeding the C method by 114.7% in the NRMSE and 136.4% in the MAAPE. Similarly, in the height trait, the A method displayed notable advantages, with improvements of 102.8% in the NRMSE and 127.8% in the MAAPE over the C method. Across all traits (ATs), the A method remained superior, boasting a substantial 119.5% reduction in the NRMSE and a significant 144.4% reduction in the MAAPE compared to the C method. See details in Table S2 (Supplementary Materials), and a visual representation of the observed and predicted values under both methods can be found in Figures S4–S7 in the Supplementary Materials.

### 3.4. Dataset 3 EYT_2 Data

Figure 3A and Figure 3C illustrate the results obtained from computing two key performance metrics, MAAPE and NRMSE, using the complete testing dataset. In this context, the Conventional method outperformed the novel method for the prediction of individual traits (DHTD, DTMT, GY, and height), as well as for predictions across all traits (ATs). Specifically, when considering the NRMSE and MAAPE within the DHTD trait, the C method presented a superiority of 47.2% and 54.1%, respectively, over the A method. Similarly, for the DTMT trait, the C method showcased advantages of 50.7% (NRMSE) and 56.8% (MAAPE) over the A method. Likewise, in the GY trait, the C method outperformed the A method by 51.3% (NRMSE) and 56.8% (MAAPE), and in the height trait, improvements of 49.9% (NRMSE) and 52.7% (MAAPE) were observed with the C method compared to the A method. Across all traits (ATs), the C method remained superior with a 50.6% reduction in the NRMSE and a 55.4% reduction in the MAAPE. See details in Table S3 in the Supplementary Materials.

In Figure 3B and Figure 3D, an analysis of the computed metrics, particularly the NRMSE and MAAPE, exclusively considering the top 20% of the testing dataset, reveals a distinct trend. Notably, the A method consistently displayed a superior performance across all evaluated traits (DHTD, DTMT, GY, and height), as well as for predictions across all traits (ATs). Specifically, within the DHTD trait, the A method showcased substantial enhancements over the C method, with reductions of 163.8% in the NRMSE and an impressive 206.3% in the MAAPE. Turning to the DTMT trait, the A method presented superiority with a notable 168.6% reduction in the NRMSE and an impressive 188.9% decrease in the MAAPE compared to the C method. Similarly, in the GY trait, the A method presented a remarkable performance lead, surpassing the C method by 89.8% in the NRMSE and 118.2% in the MAAPE. The A method also outperformed the C method in the height trait, with improvements of 88.2% in the NRMSE and 142.9% in the MAAPE. Extending this analysis to predictions across traits (ATs), the A method continued to outshine the C method, boasting substantial reductions of 114.4% in the NRMSE and 140.0% in the MAAPE. See details in Table S3. Additionally, Figures S8–S11 in the Supplementary Materials visually depict the observed and predicted values under both C and A methods.

### 3.5. Dataset 5 Maize Data

Figure 4A and Figure 4C show that when computing the two metrics (MAAPE and NRMSE) using the complete testing set, the best predictive performance was achieved using conventional methods, specifically for the GY trait. In terms of the NRMSE and MAAPE for the GY trait, the C method outperformed the A method by 46.5% and 29.2%, respectively. See details in Table S5 (Supplementary Material).

Moving on to Figure 4B and Figure 4D, when focusing on the metrics calculated exclusively from the top 20% of the testing dataset, a significant trend emerges. It is apparent that within the evaluated trait (GY), the A method showcases a considerable performance advantage over the C method. Notably, in the GY trait, the A method exhibits a substantial lead over the C method, showing improvements of 93.5% in the NRMSE and 94.0% in the MAAPE (Supplementary Material Table S5). Moreover, Figure S16 in the Supplementary Material provides a visual representation of the observed and predicted values under both the C and A methods.

### 3.6. Dataset 6 Wheat_1 Data

Figure 5A and Figure 5C clearly display that when calculating the two metrics (MAAPE and NRMSE) using the complete testing set, a superior predictive performance for the trait Y was achieved using the Conventional method. Concerning the NRMSE and MAAPE within the Y trait, the C method exhibited a superiority of 48.4% and 53.3%, respectively, over the A method, see details in Table S6.

Turning to Figure 5B and Figure 5D, a distinct trend becomes apparent when analyzing the computed metrics, specifically the NRMSE. These metrics were exclusively derived from the top 20% of the testing dataset. Within the evaluated Y trait, the A method displayed a substantial performance advantage over the C method. Notably, in the Y trait, the A method exceeded the C method by a significant margin, showing improvements of 106.9% in the NRMSE and 141.7% in the MAAPE, see details in Table S6. Additionally, Figure S17 in the Supplementary Materials presents a visual depiction of the observed and predicted values under both the A and C methods.

### 3.7. Across Data

In Figure 6A and Figure 6C, it is evident that when calculating the two metrics (MAAPE and NRMSE) across datasets and traits, the optimal predictive performance was achieved using the Conventional method. Concerning the NRMSE and MAAPE for the traits across datasets, the C method displayed superiority over the A method by 48.6% and 38.9%, respectively, see details in Table Across.

Shifting our focus to Figure 6B and Figure 6D, an analysis of the computed metrics—particularly the NRMSE—derived exclusively from the top 20% of the testing dataset reveals a significant trend. It is evident that within the evaluated traits (ATs), the A method displayed a substantial performance advantage over the C method. Notably, for the traits across datasets, the A method outperformed the C method by a substantial margin, showcasing enhancements of 108.4% in the NRMSE and 107.4% in the MAAPE, see details in Table Across data (Supplementary Materials).

## 4. Discussion

The perspective on the use of data augmentation in the realm of machine learning and data analysis has evolved significantly in recent years. Originally seen as a simple technique to artificially increase the size of training datasets, data augmentation has now emerged as a crucial tool to improve model generalization and performance. Rather than just a means of mitigating overfitting, it is increasingly regarded as a strategy to enhance the robustness and adaptability of models. This perspective shift stems from the realization that data augmentation not only introduces diversity into the training data but also enables models to learn more invariant and meaningful features from the augmented samples. As a result, data augmentation is now viewed as an integral component of the deep learning pipeline, playing a pivotal role in improving the real-world applicability and reliability of machine learning models across various domains, from computer vision to natural language processing.

Our results show that the strategy of data augmentation led to a decrease (worse performance) in the prediction accuracy of the whole testing set by 48.6% and 38.9% in terms of the NRMSE and MAAPE, respectively. It is important to note that in this study, data augmentation was only carried out on the top 20% of lines in the training set, and the training set used was this top 20% of the training plus the corresponding augmented data. However, when we observe the prediction performance on the top 20% of the testing set, we can observe that data augmentation helps to significantly increase the prediction performance of the top lines by 108.4% in the NRMSE and 107.4% in the MAAPE across traits and datasets.

These results on the use of data augmentation for genomic prediction are promising, demonstrating the potential to revolutionize the field of plant breeding. Genomic prediction relies heavily on the quality and quantity of training data, and data augmentation offers a powerful approach to enhance both aspects. By generating synthetic data points and introducing diversity into the training dataset, data augmentation enables models to capture a wider range of genetic and phenotypic variations, leading to more robust and accurate predictions. Moreover, it mitigates issues such as data scarcity and imbalances, which are common in genomics. For this reason, the expanded and enriched datasets significantly improve the generalization and reliability of genomic prediction models. In an era where precision breeding is essential to address global food security and sustainability challenges, data augmentation, according to our results, is a very promising tool to accelerate progress, drive innovation, and unlock the full potential of genomics in plant breeding.

However, its implementation is challenging since if data augmentation is carried out many times without a particular goal in mind, instead of helping to improve the prediction accuracy, it can be harmful, as observed when the performance on the whole testing set was evaluated. However, because our goal was to improve the prediction of the top lines (the more productive lines were evaluated), the training dataset consisted of only the 20% of the best lines in the training set plus the fully augmented data of this top 20% of lines. This means that overall, while data augmentation offers immense potential for the improvement of model performance and generalization, it requires careful planning, domain expertise, and quality control to ensure its successful implementation without introducing unintended issues or biases. It is crucial to emphasize that the data augmentation (DA) approach is applicable not only to the Bayesian GBLUP model but also to various other statistical machine learning models. However, implementing it optimally with other algorithms requires further research. In our study, we exclusively utilized prediction error metrics such as the mean squared error (MSE) and the mean absolute percentage error (MAAPE). Notably, we did not observe improvements in terms of Pearson’s correlation coefficient. Consequently, we encourage additional research that employs data augmentation to fine-tune more effectively, aiming not only to reduce prediction errors but also to enhance Pearson’s correlation.

One inherent limitation in our approach lies in the exclusive augmentation of the top lines within each environment during training, focusing solely on these augmented observations for the final training phase. Consequently, our data augmentation strategy disproportionately underscores the importance of these top lines, leading to a reduction in prediction errors specifically for them in the testing set. Despite this targeted improvement, our augmentation strategy falls short of optimizing the overall performance, as it fails to effectively mitigate prediction errors across the entirety of the dataset.

Furthermore, despite significant reductions in prediction errors observed for the top lines in the testing set, no corresponding enhancement was noted in terms of Pearson’s correlation for either the entire testing set or its top lines. Consequently, we advocate for further research employing data augmentation (DA) in the context of genomic prediction. The proposed approach is not deemed optimal, and the question of whether the effectiveness of data augmentation can generalize across various crops, traits, and genetic backgrounds remains unanswered. The inherent variability in genomic data may impact the suitability and results of DA techniques. Additionally, the utilization of DA, particularly within the realm of genomic selection (GS), necessitates thoughtful consideration of the synthetic data-generation methods employed. The intricacy of these methods and the requirement for domain-specific expertise to effectively apply them may constrain the accessibility and uptake of DA within GS.

We attribute the absence of an improvement in the Pearson’s correlation metrics to a phenomenon known as range restriction [24]. This occurs when the computation of metrics, such as Pearson’s correlation, is based on a restricted sample rather than the entire dataset. Consequently, we advocate for further investigation into how to fully leverage data augmentation techniques within the context of genomic prediction.

In this application, we used data augmentation techniques to enhance both the response variable (as defined in Equation (3)) and the input features (referred to as markers, as defined in Equation (2)). Specifically, we used the *mixup* method, as detailed in the Section 2. It is worth noting that numerous data augmentation methods exist; however, not all of them are suitable for tabular data, which is commonly encountered in the context of GS data. In this study, we focused exclusively on the *mixup* method, leaving ample room for the future exploration of alternative techniques and methods to fine-tune the data generated using the *mixup* approach. It is essential to emphasize the importance of a thoughtful and well-considered implementation of data augmentation techniques. This is critical to leverage the potential benefits of such methods, as there is a growing body of empirical evidence suggesting that data augmentation can significantly enhance model performance, mitigate data scarcity issues, and improve generalization, and it is continuing to evolve as a valuable tool in the toolkit of machine learning practitioners.

In general, our results provide empirical evidence that data augmentation techniques are promising tools to generate synthetic data that offer a multitude of advantages and hold significant potential across a wide spectrum of applications. Some of these advantages are (1) enhanced data privacy and security. Synthetic data generation empowers organizations to construct realistic and representative datasets without compromising the confidentiality of sensitive or private information. (2) Scalability. The process of generating synthetic data is scalable and does not require the arduous collection and manual labeling of extensive real-world datasets. (3) Data diversity. Data augmentation techniques can generate diverse data samples that encompass various scenarios and edge cases, which may prove challenging to capture through real-world data collection. (4) Mitigating data imbalances. Synthetic data generation can effectively address imbalances in datasets by generating additional samples for minority classes, thereby enhancing the overall performance of machine learning models. (5) Accelerated research. In the realm of research and experimentation, synthetic data can speed up prototyping and hypothesis testing, enabling researchers to explore novel concepts and iterate rapidly. In conclusion, the application of data augmentation to generate synthetic data stands as a promising avenue with far-reaching benefits for data-driven endeavors [13,14,15,16].

## 5. Conclusions

The GS methodology is revolutionizing plant breeding, although it still needs to guarantee highly consistent predictions to encourage its widespread application. However, this is still not the case since many factors affect its performance. Due to this, this paper explored the data augmentation approach to generate synthetic data and to increase the size and diversity of the training set. Our results show that data augmentation is a promising tool, since it consistently decreased the prediction error of the top lines in the testing set by on average 108.4% in terms of the NRMSE and 107.4% in terms of the MAAPE across traits and datasets. However, we also observed that it did not decrease the prediction error of the whole testing set, since our strategy of data augmentation only generated synthetic datasets from the top 20% of the best lines in the training set and the models were trained with only this top 20% plus the generated synthetic data from these lines. We are aware that further exploration is required regarding the best ways to take advantage of data augmentation in the context of genomic selection, but these results show that data augmentation is a very promising tool.

## Figures and Tables

**Table 1:** Summary of the fourteen datasets. GBS denotes the genotyping-by-sequencing technology and MAF denotes the minor allele frequency.

Data	Acronym	Traits	No. Lines	No. Markers	Marker Technology	MAF
Dataset 1	Disease	Pyrenophora tritici-repentis (PTR), Parastagonospora nodorum (SN), Bipolaris sorokiniana (SB)	438	11,617	GBS	0.05
Dataset 2	EYT_1	Days to heading (DTHD, number of days from germination to 50% spike emergence), days to maturity (DTMT, number of days from germination to 50% physiological maturity or the loss of green color in 50% of the spikes), plant height (height), and grain yield (GY).	766	2038	GBS	0.05
Dataset 3	EYT_2	DTHD, DTMT, height, GY	775	2038	GBS	0.05
Dataset 4	EYT_3	DTHD, DTMT, height, GY	964	2038	GBS	0.05
Dataset 5	Maize	GY	722	54,113	GBS	0.05
Dataset 6	Wheat_1	GY	1301	78,606	GBS	0.05
Dataset 7	Wheat_2	GY	1403	78,606	GBS	0.05
Dataset 8	Wheat_3	GY	1403	78,606	GBS	0.05
Dataset 9	Wheat_4	GY	1388	78,606	GBS	0.05
Dataset 10	Wheat_5	GY	1398	78,606	GBS	0.05
Dataset 11	Wheat_6	GY	1277	78,606	GBS	0.05
Dataset 12	Indica	GY = grain yield, PHR = percentage of head rice recovery, GC = percentage of chalky grains, PH = plant height	327	16,383	GBS	0.05
Dataset 13	Japonica	GY, PHR, GC, and PH	320	16,383	GBS	0.05
Dataset 14	Groundnut	Seed yield per plant (SYPP), pods per plant (NPP), pod yield per plant (PYPP), and yield per hectare (YPH).	318	8268	GBS	0.05

## Data Availability

The complete datasets are available at: https://github.com/osval78/Data_Augmentation_Regression (accessed on 1 January 2020).

## Supplementary Materials

The following supporting information can be downloaded at: https://www.mdpi.com/article/10.3390/genes15030286/s1. This section contains the results from all 14 data sets. Results from data sets 1, 2, 3, 5, and 6 were already discussed in the main text. Supplementary Material also provides detail results from the other 9 data sets: Dataset 4 (EYT_3) (Table S4 and Figures S12–S15); Dataset 7 (Wheat_2 (Table S7 and Figure S18); Dataset 8 (Wheat_3) (Table S8 and Figure S19); Dataset 9 (Wheat_4) (Table S9 and Figure S20); Dataset 10 (Wheat_5) (Table S10 and Figure S21); Dataset 11 (Wheat_6) (Table S11 and Figure S22); Dataset 12 (Indica) (Table S12 and Figures S23–S26); Dataset 13 (Japonica) (Table S13 and Figures S27–S30); Dataset 14 (Groundnut) (Table S14 and Figures S31–S34).
