# Supplementary material for: Data Augmentation Enhances Plant-Genomic-Enabled Predictions
Source: Genes (Basel). 2024 Feb 24;15(3):286. doi: 10.3390/genes15030286 (PMC10969940; doi:10.3390/genes15030286)
Supplement: Supplementary file 1 [file genes-15-00286-s001.zip › genes-2839092-supplementary.pdf]

SUPPLEMENTARY MATERIAL

Dataset 1 Disease

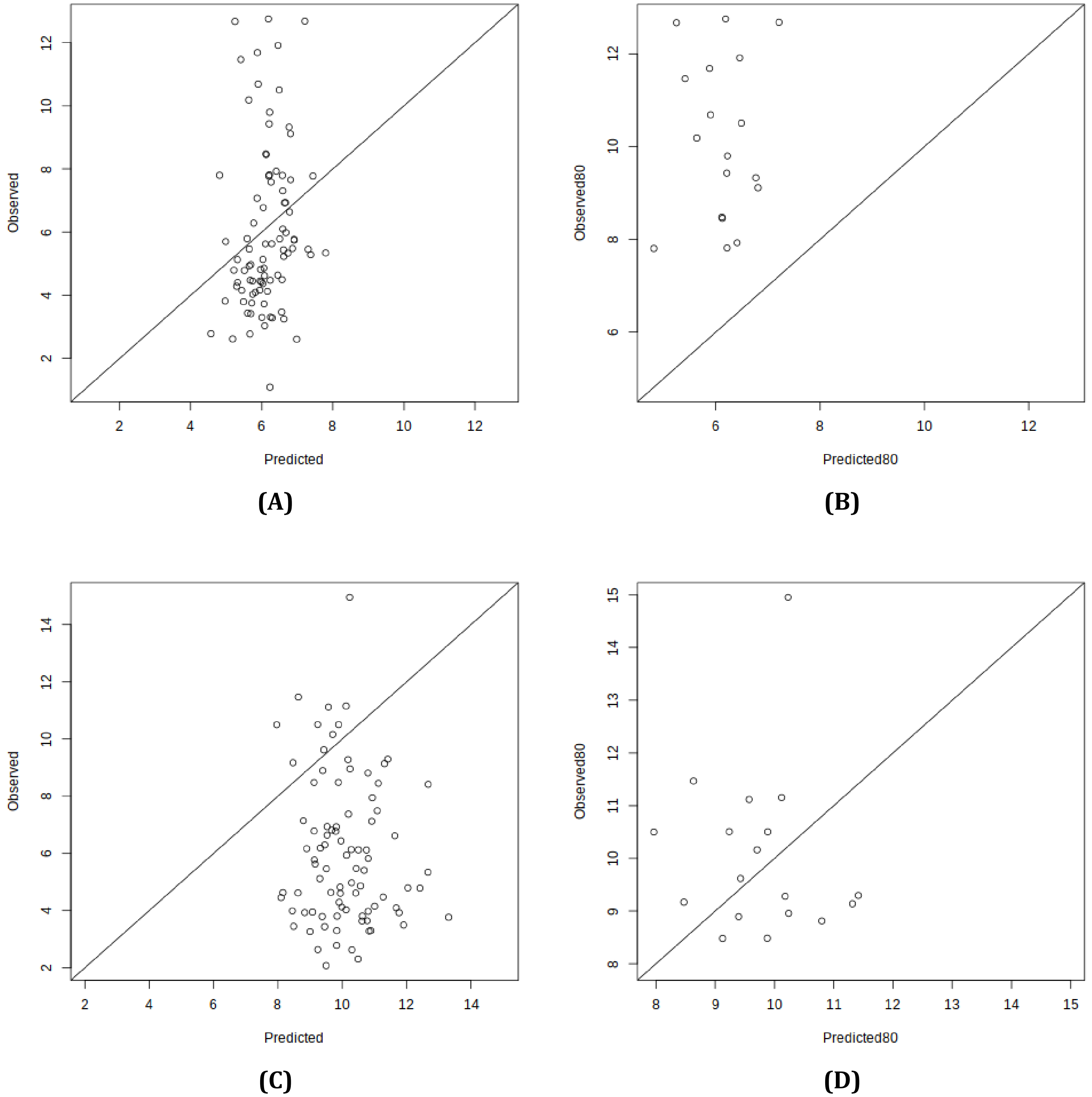

Figure S1. Prediction performance results for Dataset 1 (**Disease**) in the trait **PTR**, using the Conventional and Augmented methods, in terms of (**A**) the plots generated for the total testing using the Conventional method, (**B**) plots generated for the top 20% of testing using the Conventional method, (**C**) the plots generated for the total testing using the Augmented method, (**D**) the plots generated for the top 20% of testing using the Augmented method.

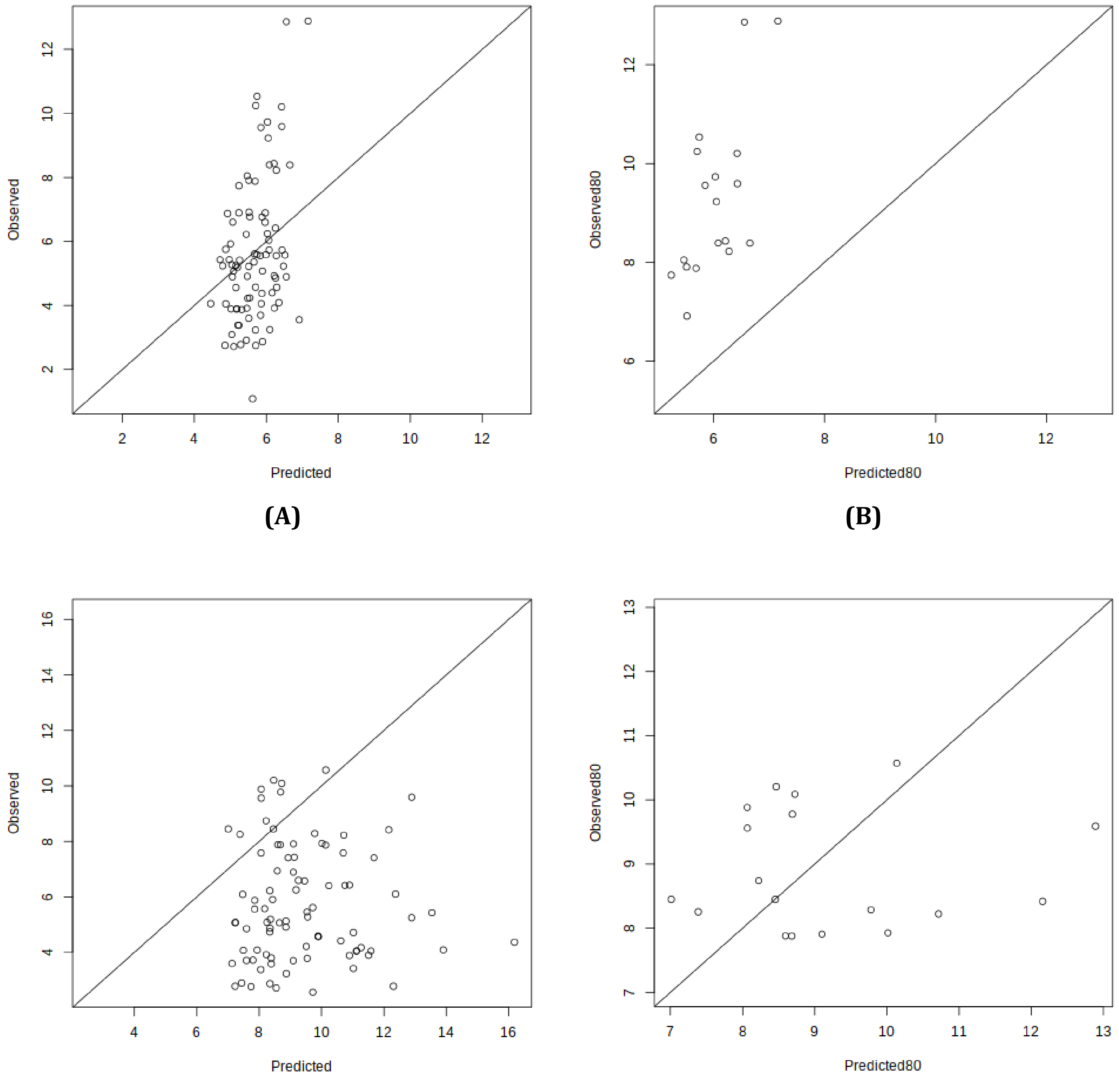

Figure S2. Prediction performance results for dataset Dataset 1 (**Disease**) in the trait **SB**, using the Conventional and Augmented methods, in terms of (**A**) the plots generated for the total testing using the Conventional method, (**B**) the plots generated for the top 20% of testing using the Conventional method, (**C**) the plots generated for the total testing using the Augmented method, (**D**) the plots generated for the top 20% of testing using the Augmented method.

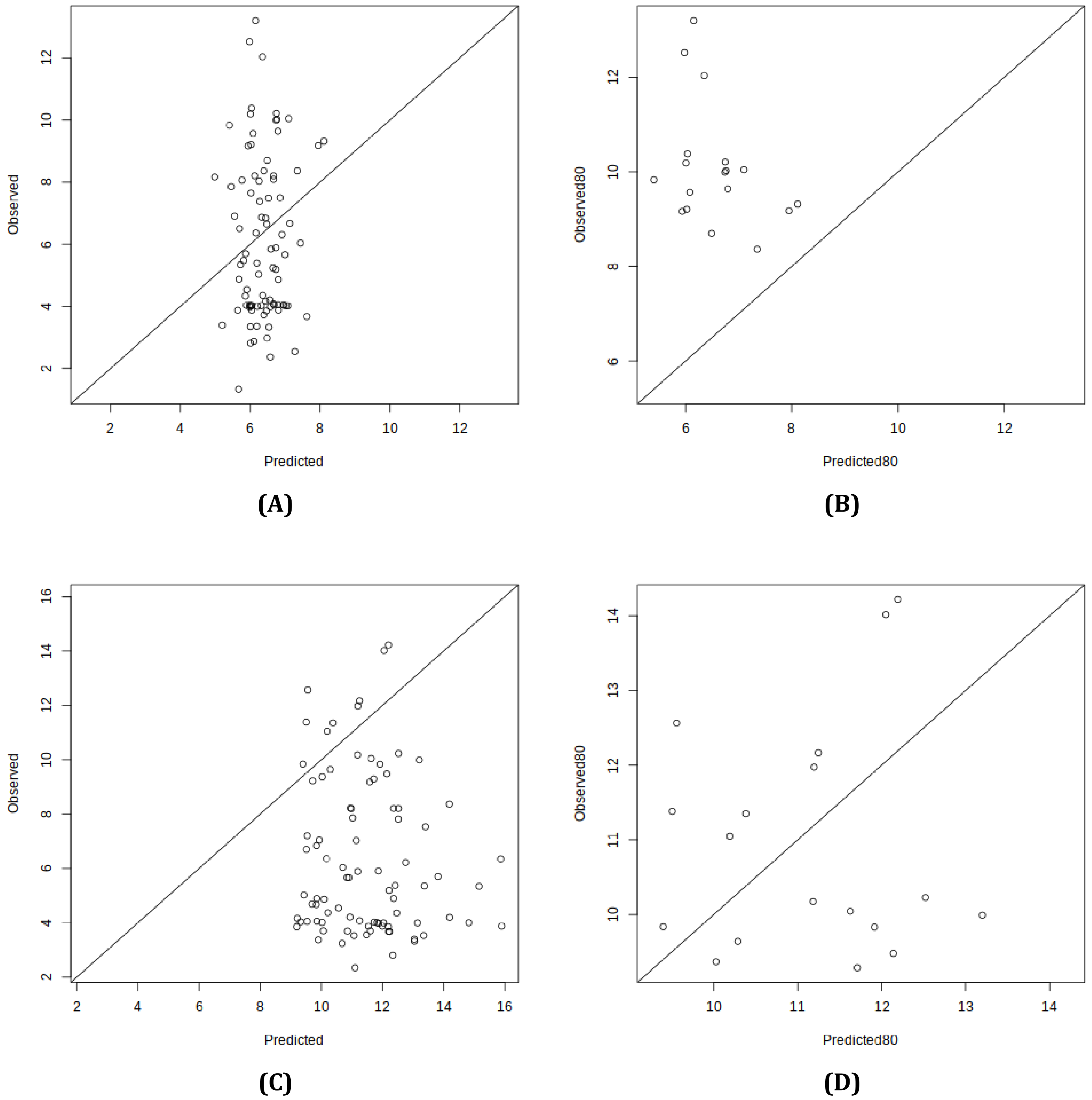

Figure S3. Prediction performance results for dataset Dataset 1 (**Disease**) in the trait **SN**, using the Conventional and Augmented methods, in terms of (**A**) the plots generated for the total testing using the Conventional method, (**B**) the plots generated for the top 80% of testing using the Conventional method, (**C**) the plots generated for the total testing using the Augmented method, (**D**) the plots generated for the top 20% of testing using the Augmented method.

Table S1. Prediction Accuracy Results for Dataset 1 (**Disease**) using the C and A Methods, with Metrics MAAPE and NRMSE for the total testing, and MAAPE and NRMSE for the top 20% testing.

Dataset	**Trait**	**Method**	**NRMSE**	**MAAPE**	**NRMSE_80**	**MAAPE_80**
Disease	PTR	C	0.975	0.334	2.933	0.359
Disease	PTR	A	1.880	0.636	1.189	0.142
Disease	SB	C	0.974	0.289	2.156	0.307
Disease	SB	A	1.971	0.599	1.497	0.174
Disease	SN	C	1.002	0.378	3.056	0.377
Disease	SN	A	2.037	0.726	1.246	0.157
Disease	AT	C	0.983	0.334	2.715	0.347
Disease	AT	A	1.963	0.654	1.311	0.158

Dataset 2 EYT_1

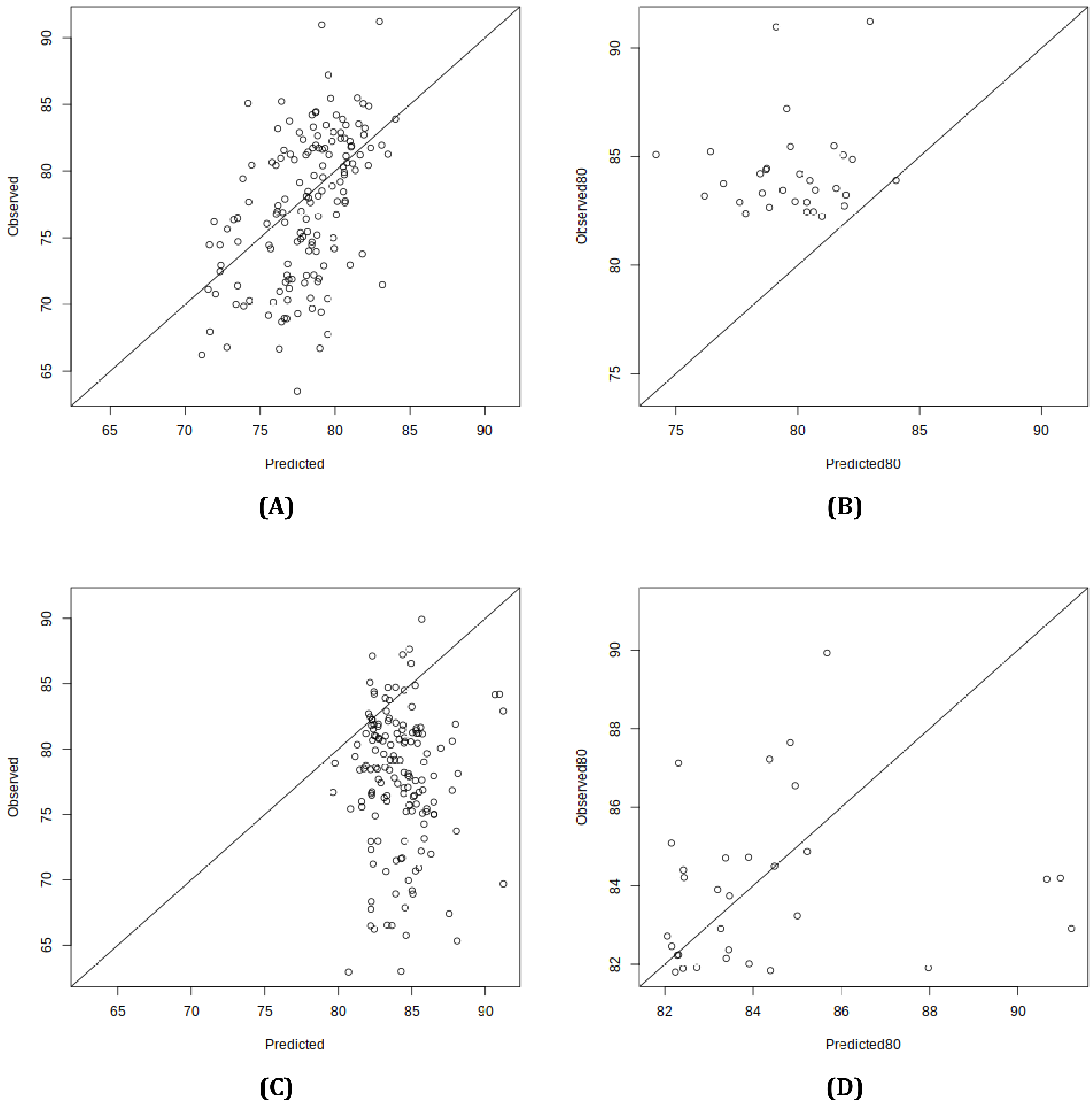

Figure S4. Prediction performance results for Dataset 2 (**EYT_1**) in the trait **DHTD**, using the Conventional and Augmented methods, in terms of (**A**) the plots generated for the total testing using the Conventional method, (**B**) the plots generated for the top 20% of testing using the Conventional method,

(**C**) the plots generated for the total testing using the Augmented method, (**D**) the plots generated for the top 20% of testing using the Augmented method.

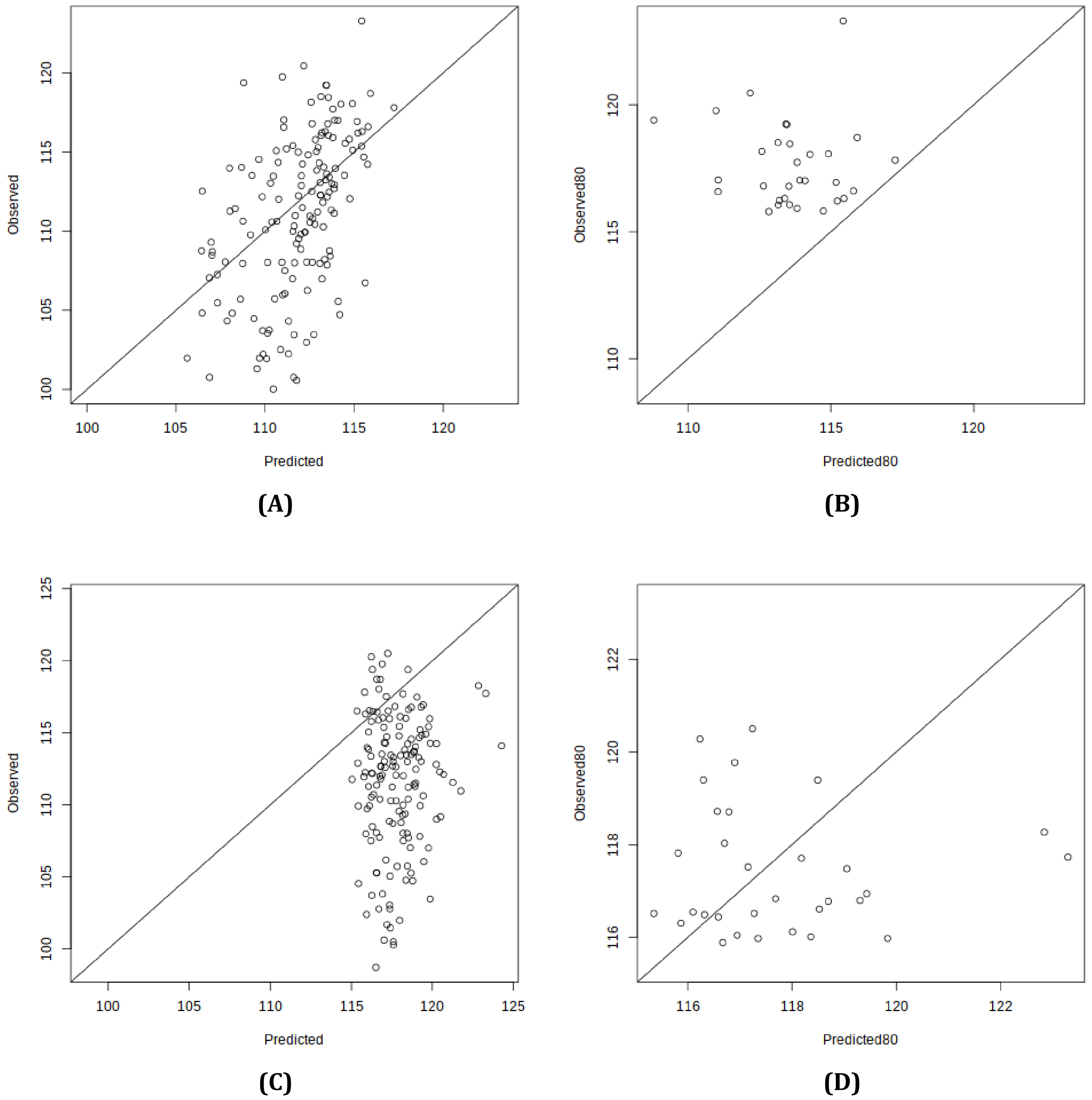

Figure S5. Prediction performance results for dataset Dataset 2 (**EYT_1**) in the trait **DTMT**, using the Conventional and Augmented methods, in terms of (**A**) the plots generated for the total testing using the Conventional method, (**B**) the plots generated for the top 20% of testing using the Conventional method, (**C**) the plots generated for the total testing using the Augmented method, (**D**) the plots generated for the top 20% of testing using the Augmented method.

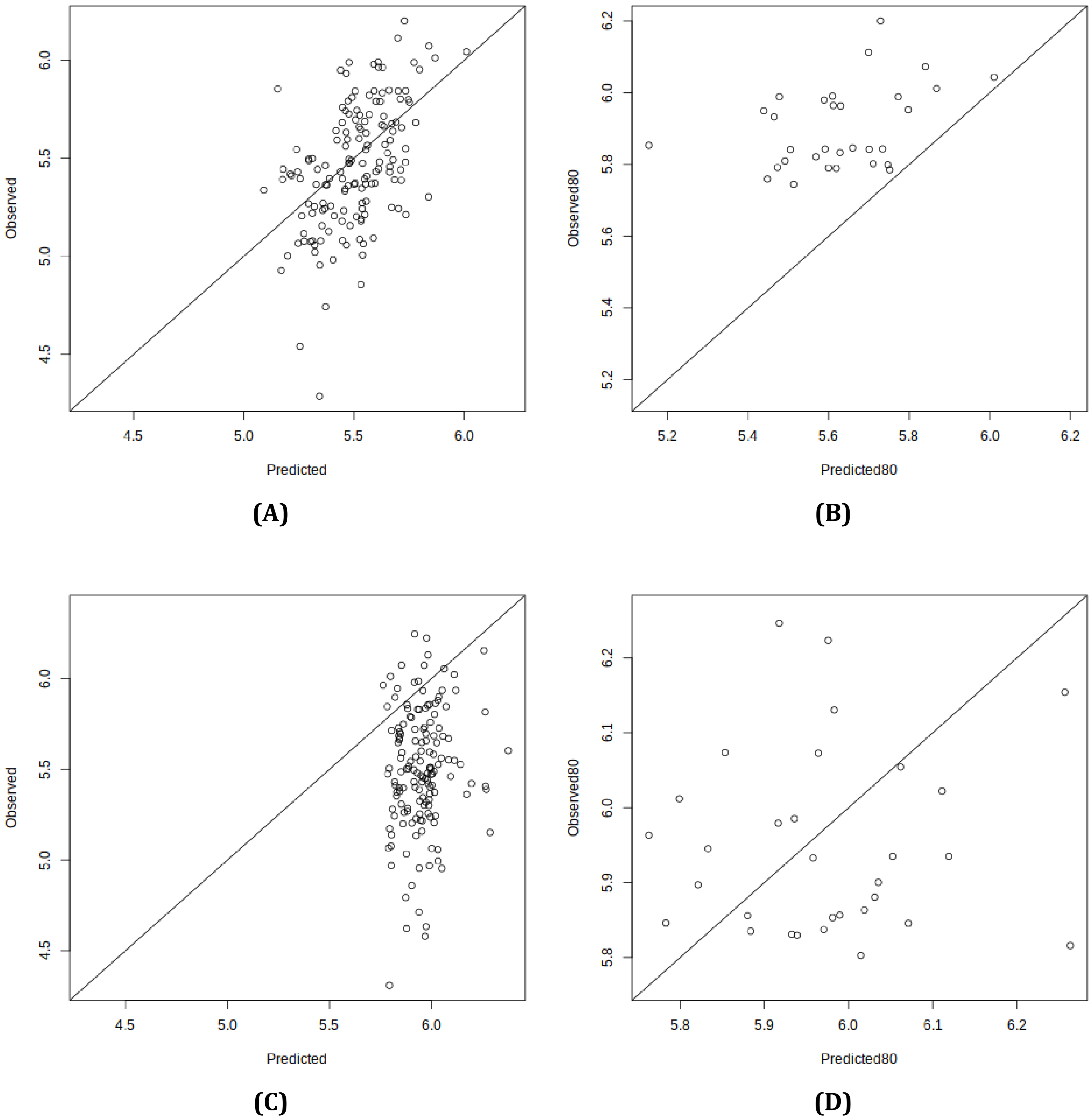

Figure S6. Prediction accuracy performance results for dataset Dataset 2 (**EYT_1**) in the trait **GY**, using the Conventional and Augmented methods, in terms of (**A**) the plots generated for the total testing using the Conventional method, (**B**) the plots generated for the top 20% of testing using the Conventional method, (**C**) the plots generated for the total testing using the Augmented method, (**D**) the plots generated for the top 20% of testing using the Augmented method.

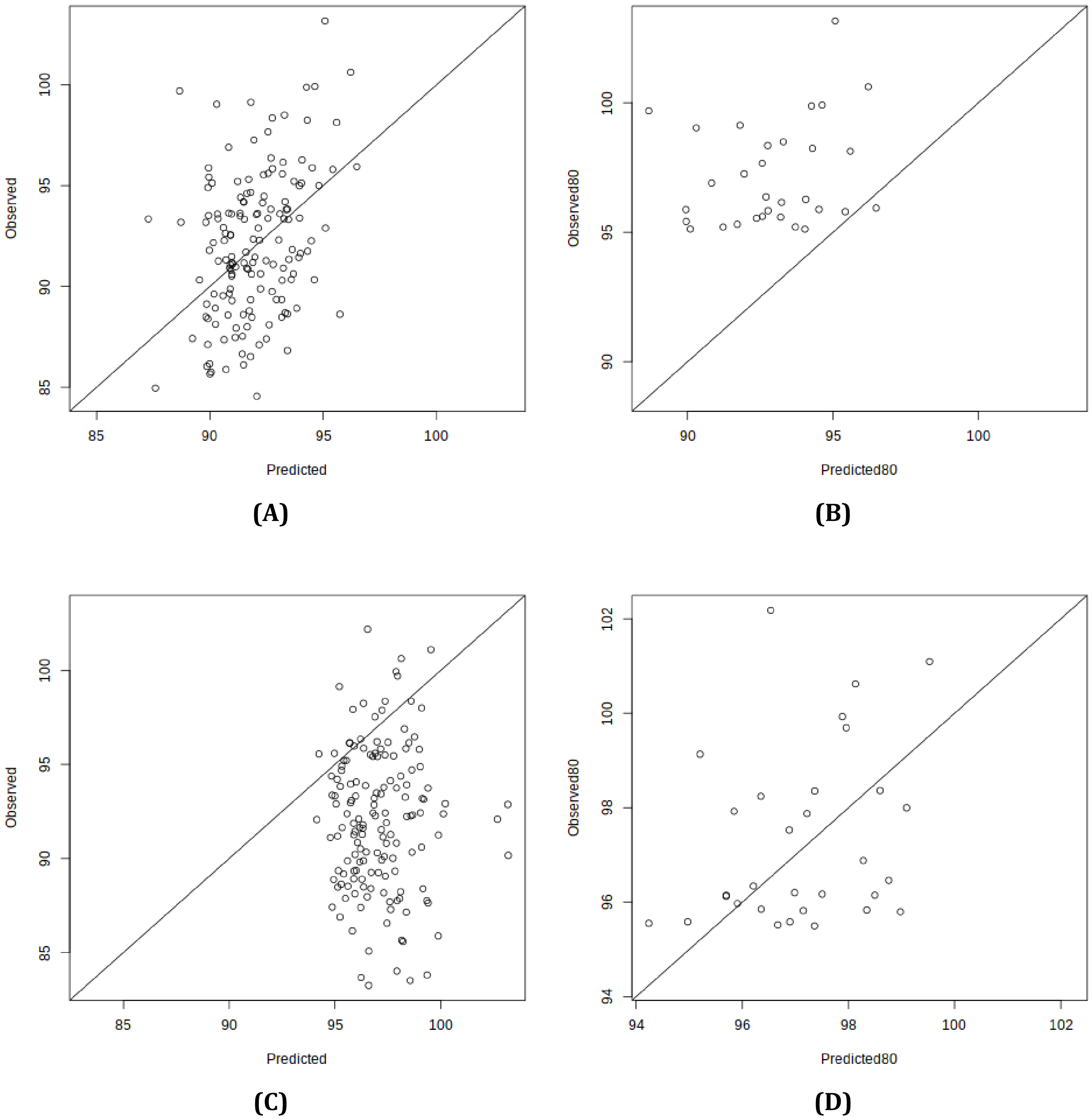

Figure S7. Prediction performance results for dataset Dataset 2 (**EYT_1**) in the trait **Height**, using the Conventional and Augmented methods, in terms of (**A**) the plots generated for the total testing using the Conventional method, (**B**) the plots generated for the top 20% of testing using the Conventional method, (**C**) the plots generated for the total testing using the Augmented method, (**D**) the plots generated for the top 20% of testing using the Augmented method.

Table S2. Prediction Accuracy Results for Dataset 2 (**EYT_1**) using the C and A Methods, with Metrics MAAPE and NRMSE for the total testing, and MAAPE and NRMSE for the top 20% testing.

Dataset	**Trait**	**Method**	**NRMSE**	**MAAPE**	**NRMSE_80**	**MAAPE_80**
EYT_1	DTHD	C	0.880	0.044	3.209	0.059
EYT_1	DTHD	A	1.619	0.088	1.294	0.023
EYT_1	DTMT	C	0.878	0.028	3.318	0.039
EYT_1	DTMT	A	1.621	0.056	1.38	0.014
EYT_1	GY	C	0.877	0.042	2.796	0.052
EYT_1	GY	A	1.713	0.089	1.302	0.022
EYT_1	Height	C	0.917	0.028	2.576	0.041
EYT_1	Height	A	1.742	0.060	1.270	0.018
EYT_1	AT	C	0.891	0.033	2.897	0.044
EYT_1	AT	A	1.692	0.068	1.320	0.018

Dataset 3 EYT_2

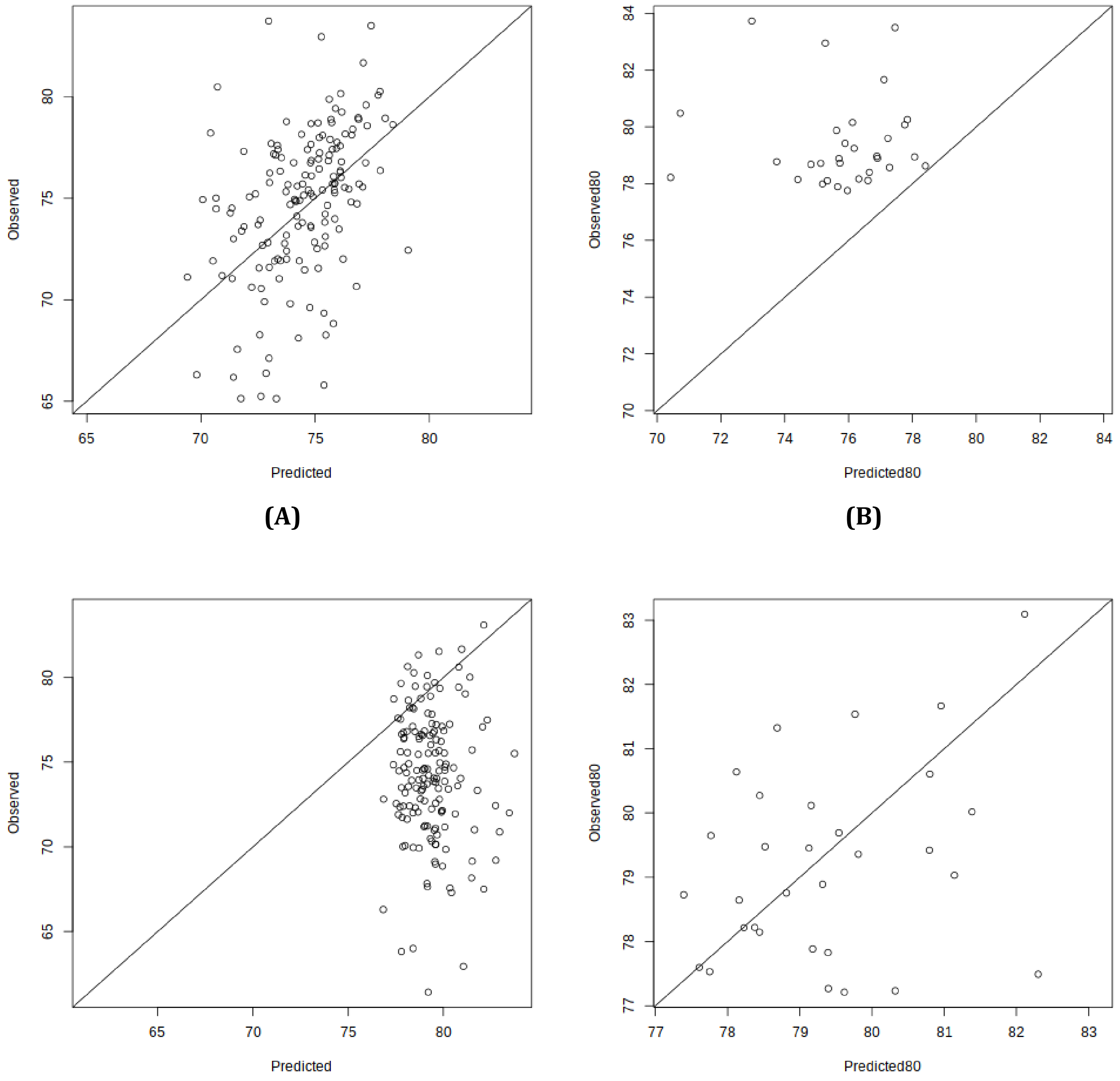

(A) **(B)** **(C)** **(D)**

Figure S8. Prediction performance results for Dataset 3 (**EYT_2**) in the trait **DHTD**, using the Conventional and Augmented methods, in terms of (**A**) the plots generated for the total testing using the Conventional method, (**B**) the plots generated for the top 20% of testing using the Conventional method, (**C**) the plots generated for the total testing using the Augmented method, (**D**) the plots generated for the top 20% of testing using the Augmented method.

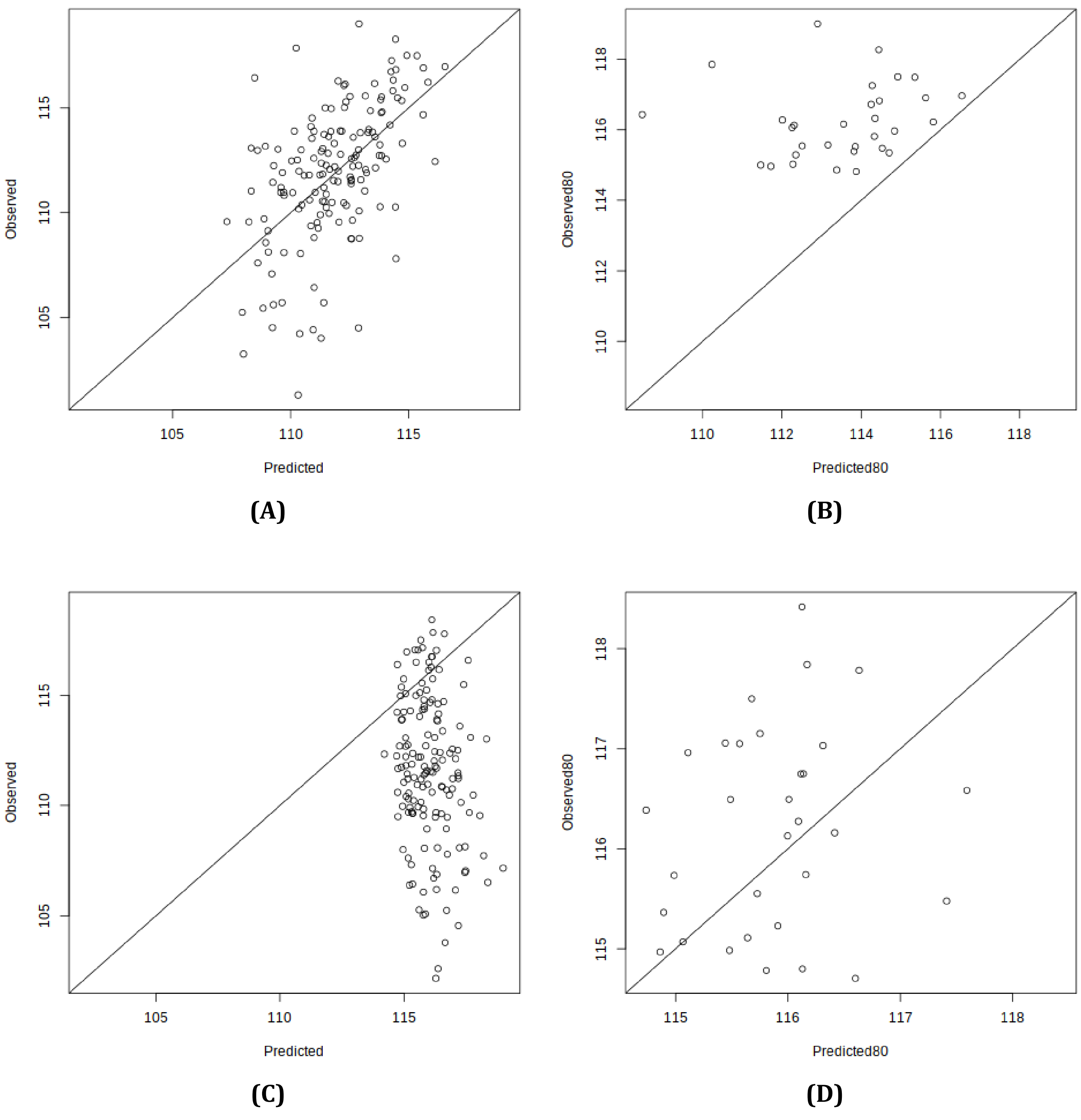

Figure S9. Prediction performance results for dataset Dataset 3 (**EYT_2**) in the trait **DTMT**, using the Conventional and Augmented methods, in terms of (**A**) the plots generated for the total testing using the Conventional method, (**B**) plots generated for the top 20% of testing using the Conventional method, (**C**) the plots generated for the total testing using the Augmented method, (**D**) the plots generated for the top 20% of testing using the Augmented method.

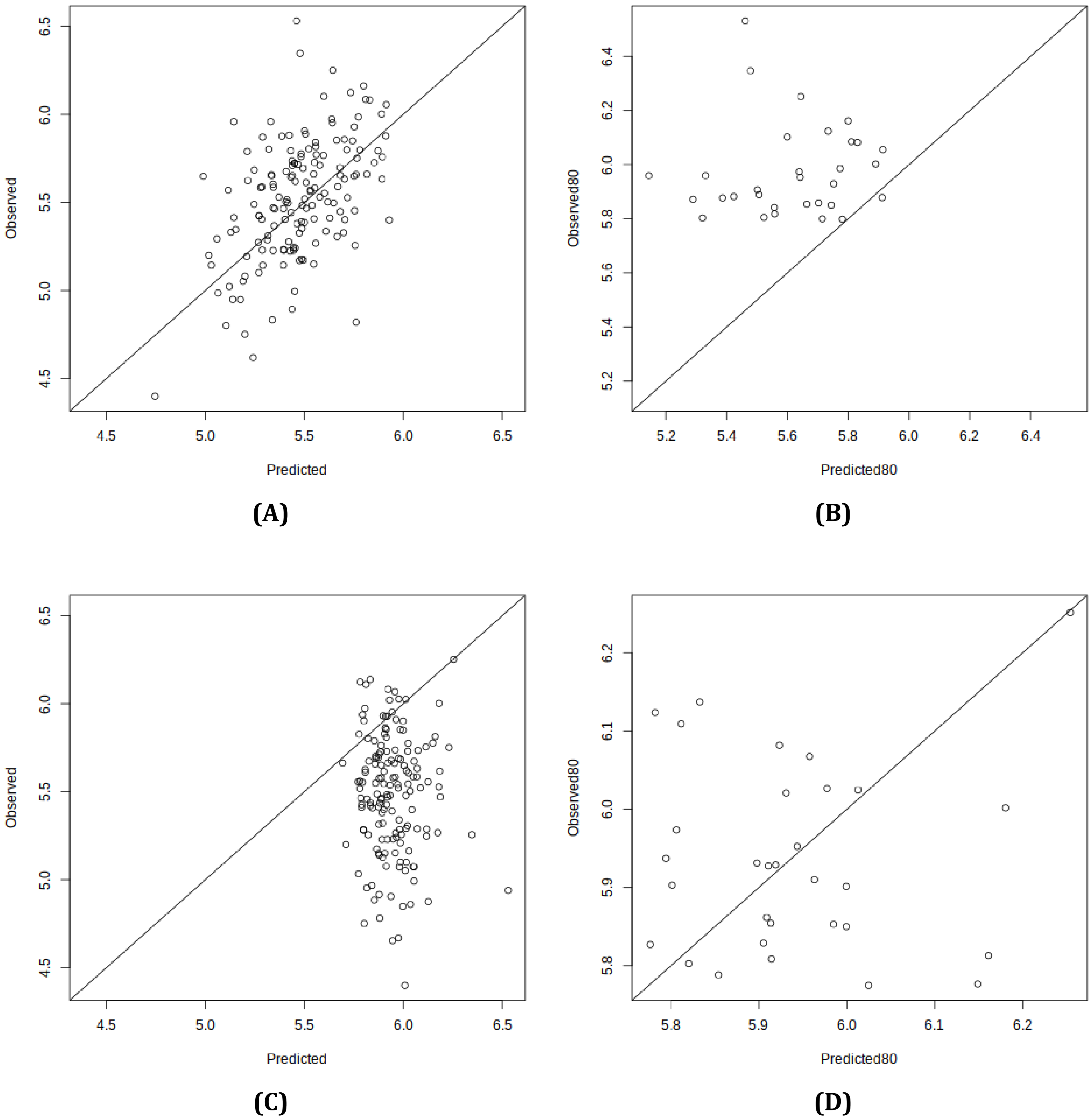

Figure S10. Prediction accuracy performance results for Dataset 3 (**EYT_2**) in the trait **GY**, using the Conventional and Augmented methods, in terms of (**A**) the plots generated for the total testing using the Conventional method, (**B**) the plots generated for the top 20% of testing using the Conventional method, (**C**) the plots generated for the total testing using the Augmented method, (**D**) the plots generated for the top 20% of testing using the Augmented method.

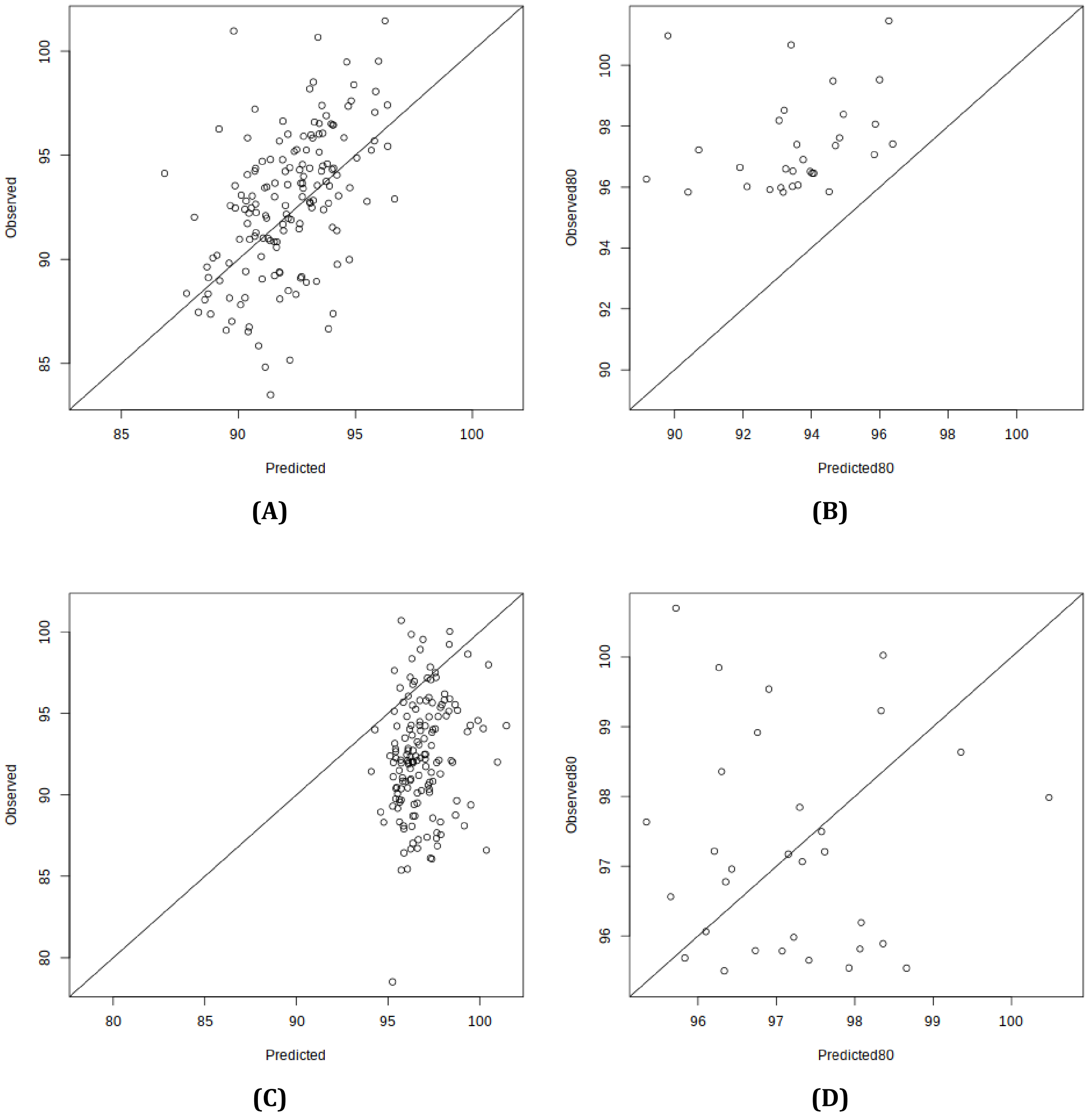

Figure S11. Prediction accuracy performance results for Dataset 3 (**EYT_2**) in the trait **Height**, using the Conventional and Augmented methods, in terms of (**A**) the plots generated for the total testing using the Conventional method, (**B**) the plots generated for the top 20% of testing using the Conventional method, (**C**) the plots generated for the total testing using the Augmented method, (**D**) the plots generated for the top 20% of testing using the Augmented method.

Table S3. Prediction Accuracy Results for Dataset **EYT_2** using the C and A Methods, with Metrics MAAPE and NRMSE for the total testing, and MAAPE and NRMSE for the top 20% testing.

Dataset	**Trait**	**Method**	**NRMSE**	**MAAPE**	**NRMSE_80**	**MAAPE_80**
EYT_2	DTHD	C	0.897	0.034	3.245	0.049
EYT_2	DTHD	A	1.699	0.074	1.230	0.016
EYT_2	DTMT	C	0.848	0.019	3.363	0.026
EYT_2	DTMT	A	1.719	0.044	1.252	0.009
EYT_2	GY	C	0.837	0.041	2.525	0.048
EYT_2	GY	A	1.717	0.095	1.330	0.022
EYT_2	Height	C	0.858	0.026	2.526	0.034
EYT_2	Height	A	1.711	0.055	1.342	0.014
EYT_2	AT	C	0.848	0.029	2.805	0.036
EYT_2	AT	A	1.716	0.065	1.308	0.015

Dataset 4 EYT_3

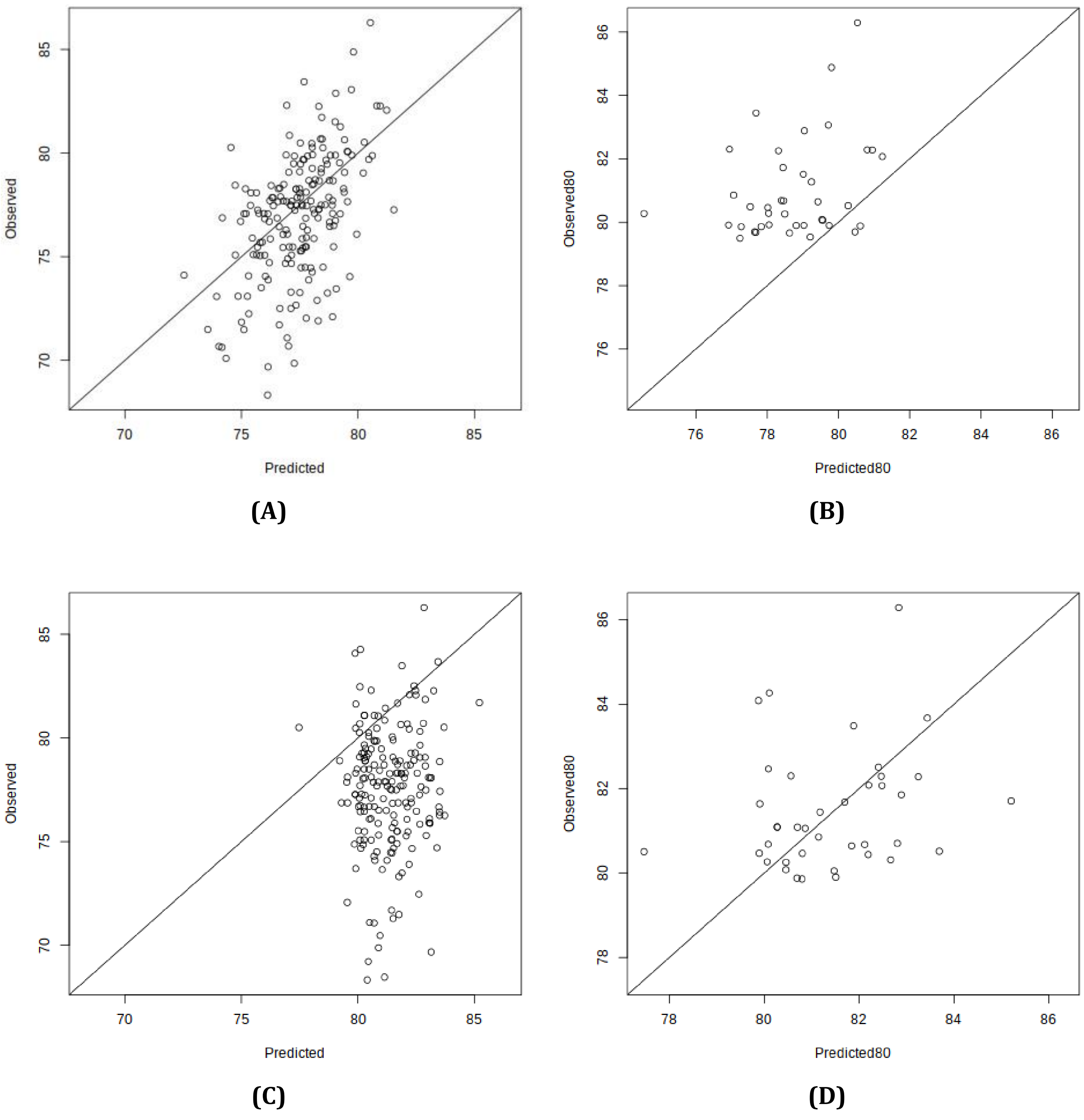

Figure S12. Prediction performance results for Dataset 4 **EYT_3** in the trait **DTHD**, using the Conventional and Augmented methods, in terms of (**A**) the plots generated for the total testing using the Conventional method, (**B**) the plots generated for the top 20% of testing using the Conventional method, (**C**) the plots generated for the total testing using the Augmented method, (**D**) the plots generated for the top 20% of testing using the Augmented method.

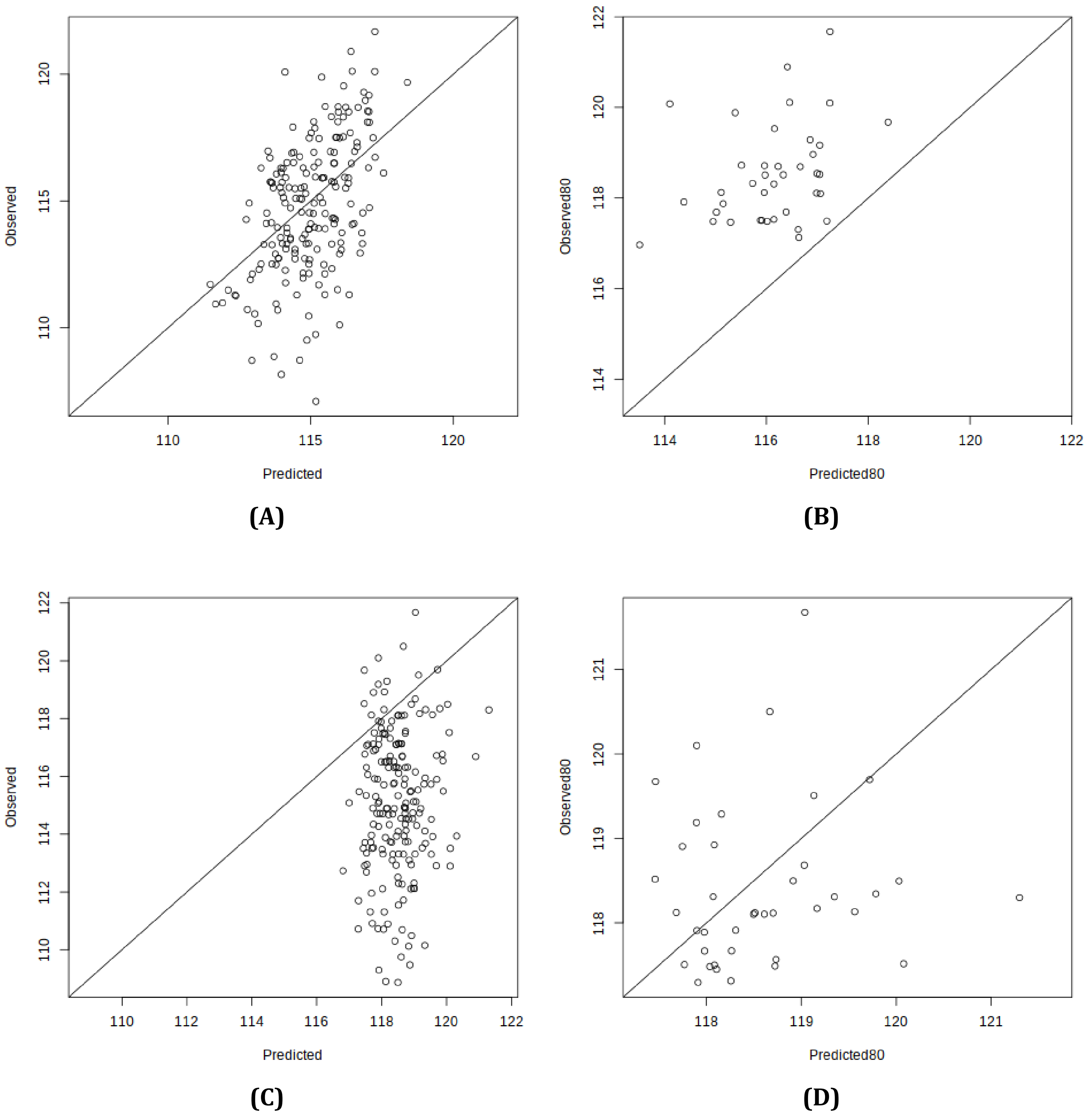

Figure S13. Prediction accuracy performance results for Dataset 4 **EYT_3** in the trait **DTMT**, using the Conventional and Augmented methods, in terms of (**A**) the plots generated for the total testing using the Conventional method, (**B**) the plots generated for the top 20% of testing using the Conventional method, (**C**) the plots generated for the total testing using the Augmented method, (**D**) the plots generated for the top 20% of testing using the Augmented method.

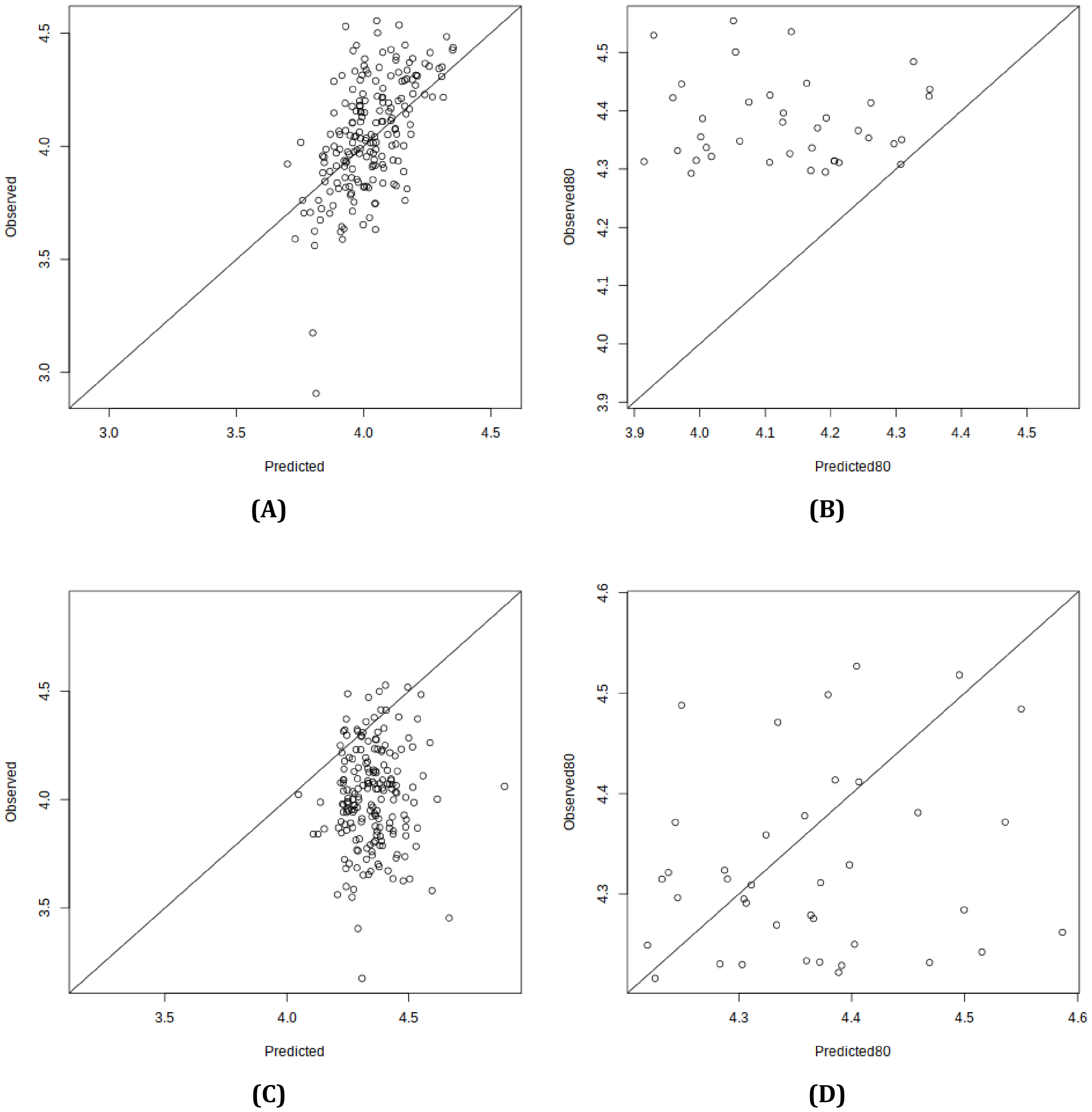

Figure S14. Prediction performance results for Dataset 4 **EYT_3** in the trait **GY**, using the Conventional and Augmented methods, in terms of (**A**) the plots generated for the total testing using the Conventional method, (**B**) the plots generated for the top 20% of testing using the Conventional method, (**C**) the plots generated for the total testing using the Augmented method, (**D**) the plots generated for the top 20% of testing using the Augmented method.

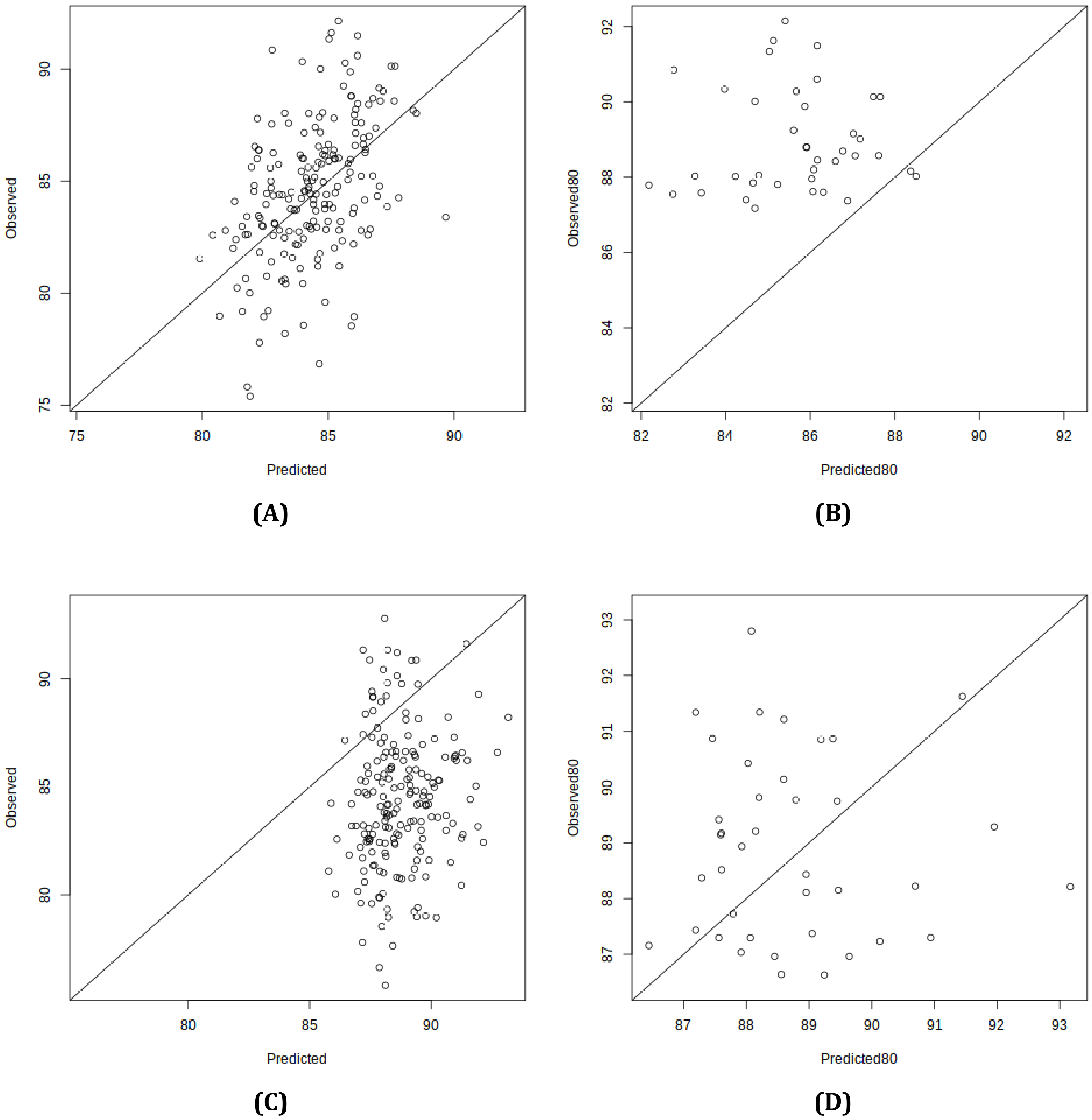

Figure S15. Prediction accuracy performance results for Dataset4 **EYT_3** in the trait **Height**, using the Conventional and Augmented methods, in terms of (**A**) the plots generated for the total testing using the Conventional method, (**B**) plots generated for the top 20% of testing using the Conventional method, (**C**) plots generated for the total testing using the Augmented method, (**D**) the plots generated for the top 20% of testing using the Augmented method.

Table S4. Prediction Accuracy Results for Dataset 4 **EYT_3** using the C and A Methods, with Metrics MAAPE and NRMSE for the total testing, and MAAPE and NRMSE for the top 20% testing.

Dataset	**Trait**	**Method**	**NRMSE**	**MAAPE**	**NRMSE_80**	**MAAPE_80**
EYT_3	DTHD	C	0.887	0.027	2.344	0.036
EYT_3	DTHD	A	1.665	0.057	1.279	0.018
EYT_3	DTMT	C	0.873	0.015	3.252	0.021
EYT_3	DTMT	A	1.686	0.033	1.311	0.007
EYT_3	GY	C	0.868	0.040	2.607	0.052
EYT_3	GY	A	1.730	0.089	1.248	0.022
EYT_3	Height	C	0.867	0.024	2.467	0.034
EYT_3	Height	A	1.719	0.055	1.454	0.018
EYT_3	AT	C	0.869	0.027	2.775	0.035
EYT_3	AT	A	1.712	0.059	1.338	0.016

Dataset 5 Maize.

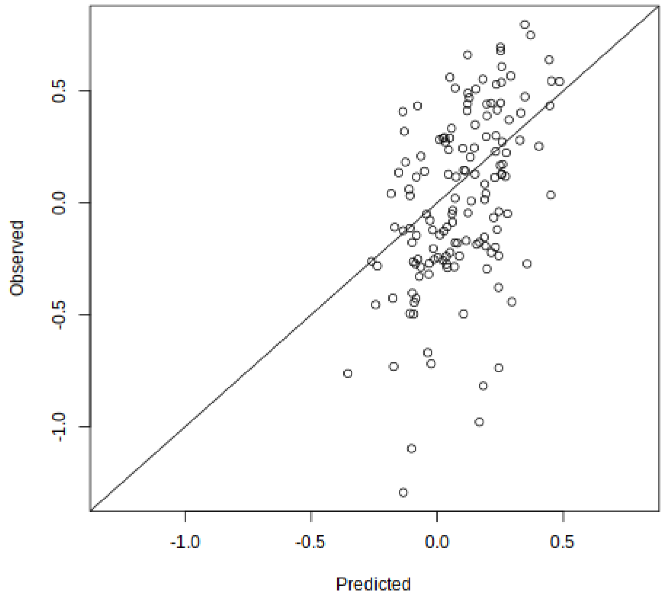

(A)

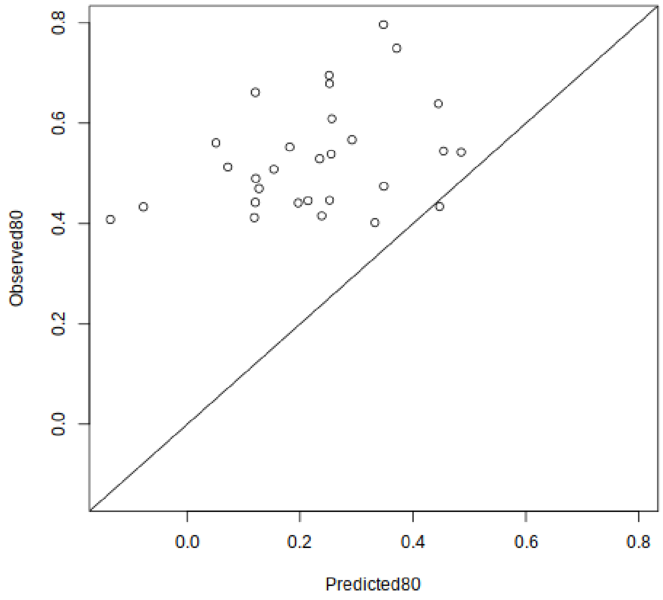

(B)

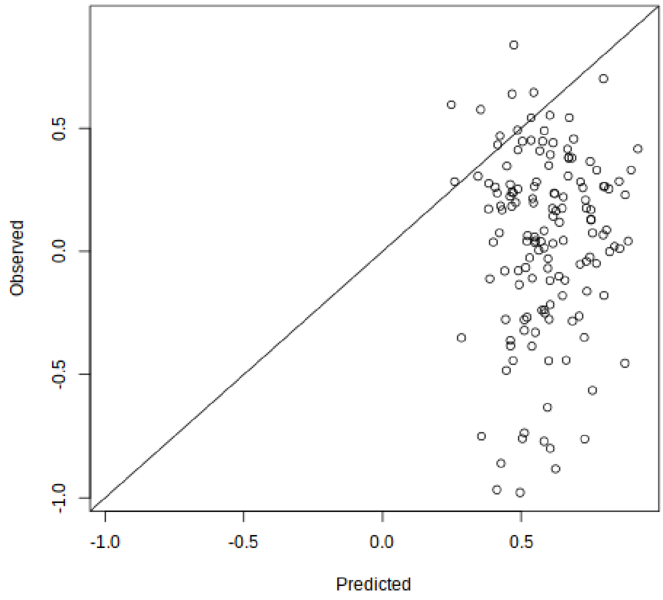

(C)

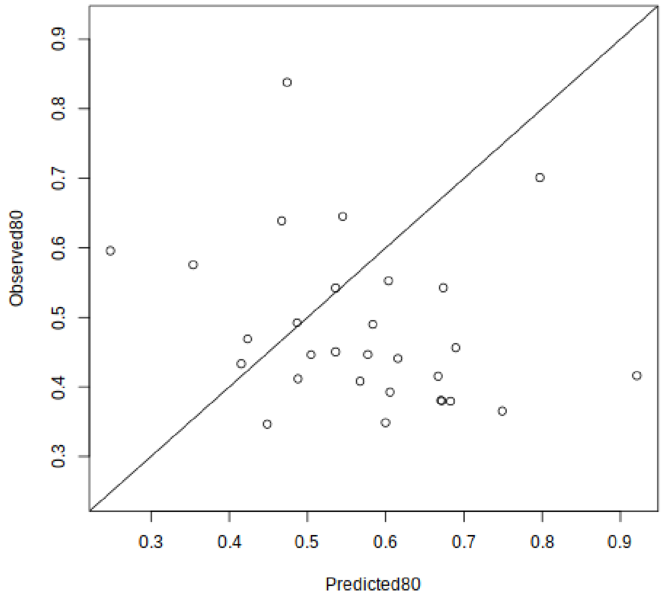

(D)

Figure S16. Prediction performance results for Dataset 5 **Maize** in the trait **GY**, using the Conventional and Augmented methods, in terms of (**A**) the plots generated for the total testing using the Conventional method, (**B**) the plots generated for the top 20% of testing using the Conventional method, (**C**) the plots

generated for the total testing using the Augmented method, (**D**) the plots generated for the top 20% of testing using the Augmented method.

Table S5. Prediction Accuracy Results for Dataset 5 **Maize** using the C and A Methods, with Metrics MAAPE and NRMSE for the total testing, and MAAPE and NRMSE for the top 20% testing.

Dataset	**Trait**	**Method**	**NRMSE**	**MAAPE**	**NRMSE_80**	**MAAPE_80**
Maize	GY	C	0.902	0.716	2.518	0.578
Maize	GY	A	1.686	1.011	1.301	0.298

Dataset 6 Wheat_1

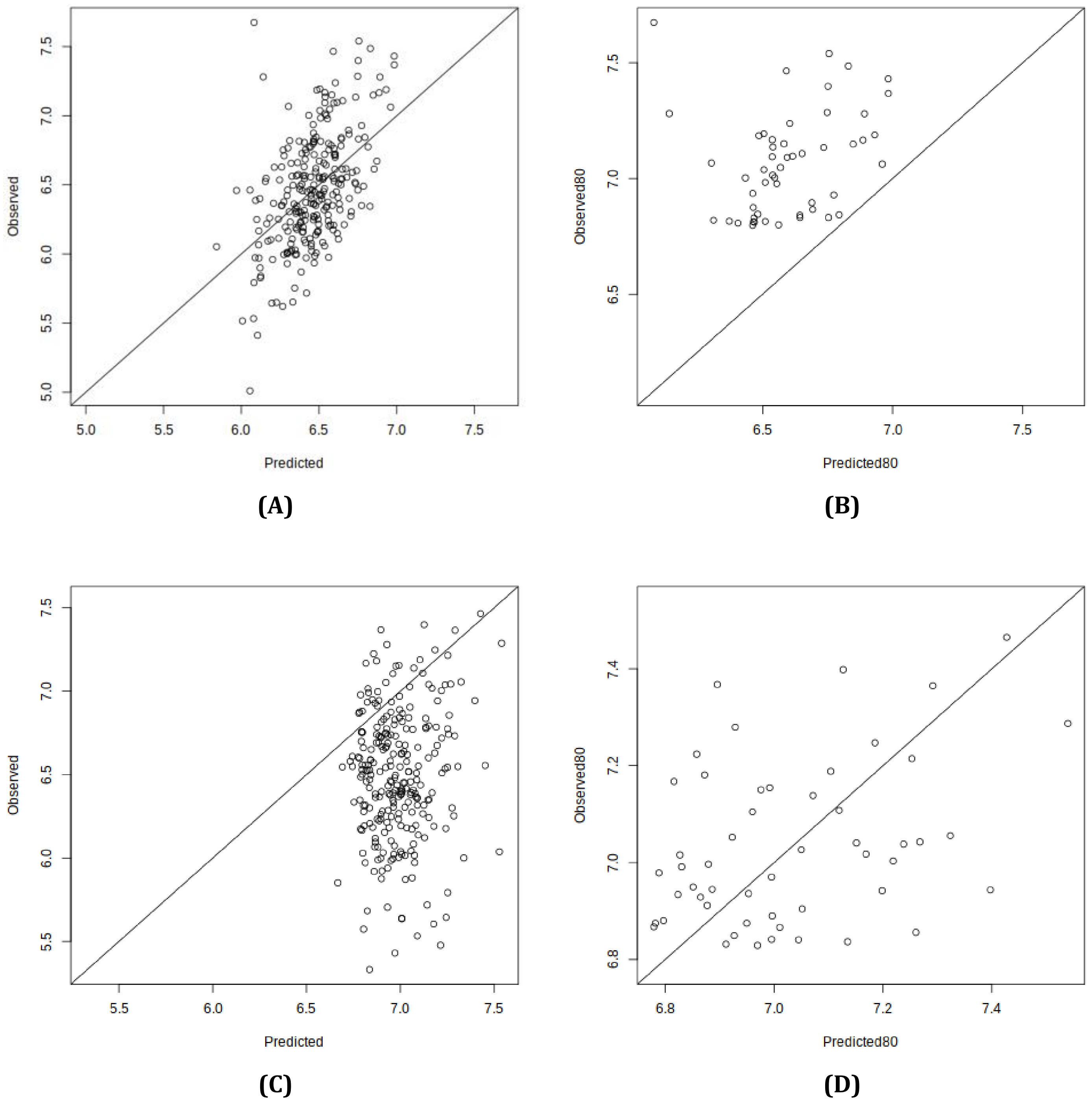

Figure S17. Prediction performance results for Dataset 6 **Wheat_1** in the trait **Y**, using the Conventional and Augmented methods, in terms of (**A**) the plots generated for the total testing using the Conventional method, (**B**) plots generated for the top 20% of testing using the Conventional method, (**C**) the plots generated for the total testing using the Augmented method, (**D**) the plots generated for the top 20% of testing using the Augmented method.

Table S6. Prediction Accuracy Results for Dataset 6 **Wheat_1** using the C and A Methods, with Metrics MAAPE and NRMSE for the total testing, and MAAPE and NRMSE for the top 20% testing.

Dataset	**Trait**	**Method**	**NRMSE**	**MAAPE**	**NRMSE_80**	**MAAPE_80**
Wheat_1	Y	C	0.880	0.042	2.491	0.058
Wheat_1	Y	A	1.704	0.090	1.204	0.024

Across data .

Table Across. Average Prediction Accuracy Results for Dataset **Across_Data** using the C and A Methods, with Metrics MAAPE and NRMSE for the total testing, and MAAPE and NRMSE for the top 20% testing (NRMSE_80 and MAAPE_80)

Dataset	**Trait**	**Method**	**NRMSE**	**MAAPE**	**NRMSE_80**	**MAAPE_80**
Across_Data	AT	C	0.900	0.230	2.685	0.212
Across_Data	AT	A	1.752	0.377	1.288	0.102

Dataset 7 Wheat_2

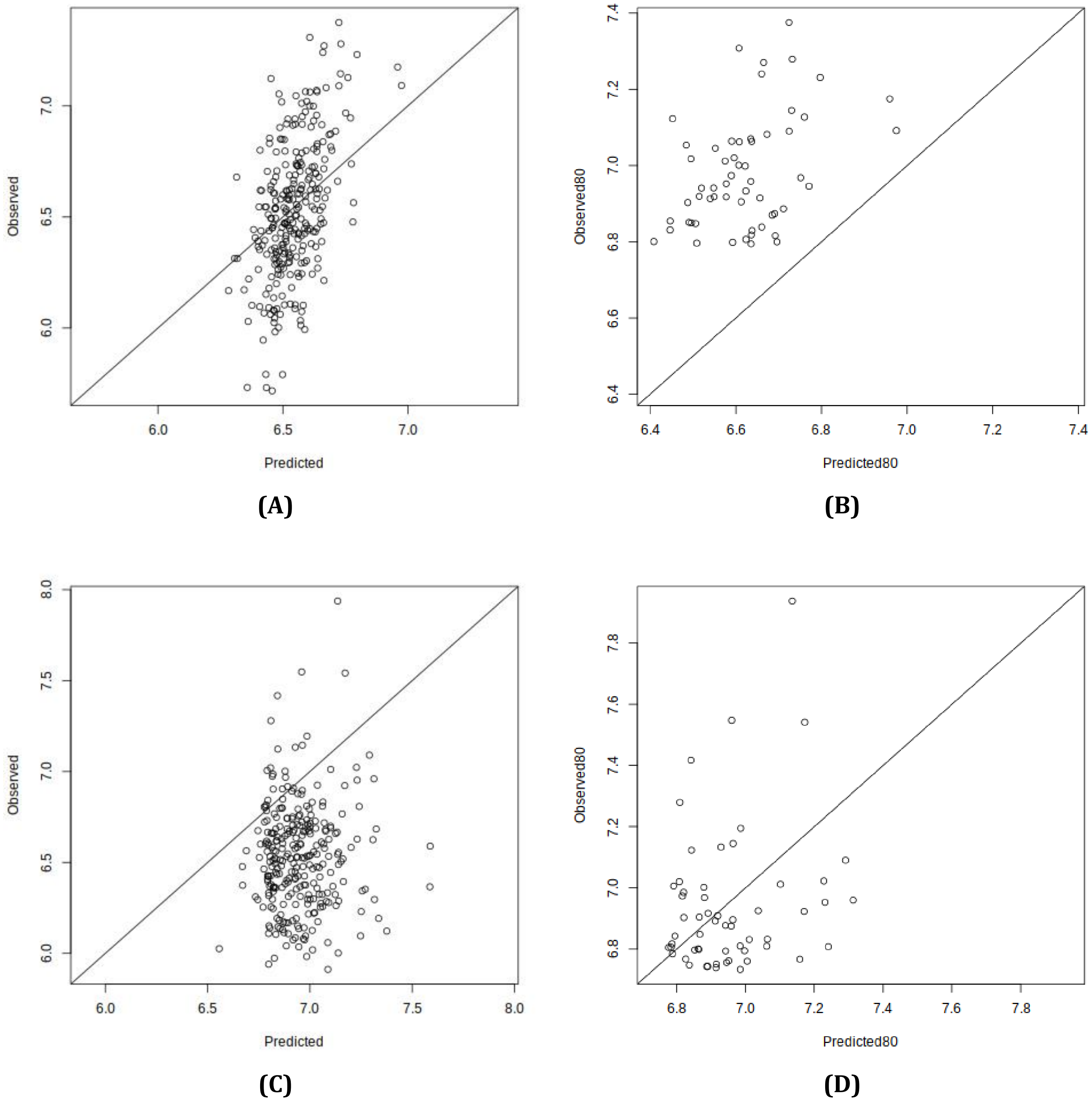

Figure S18. Prediction performance results for Dataset 7 **Wheat_2** in the trait **Y**, using the Conventional and Augmented methods, in terms of (**A**) the plots generated for the total testing using the Conventional method, (**B**) the plots generated for the top 20% of testing using the Conventional method, (**C**) the plots generated for the total testing using the Augmented method, (**D**) the plots generated for the top 20% of testing using the Augmented method.

Table S7. Prediction Accuracy Results for Dataset 7 **Wheat_2** using the C and A Methods, with Metrics MAAPE and NRMSE for the total testing, and MAAPE and NRMSE for the top 20% testing.

Dataset	Trait	Method	NRMSE	MAAPE	NRMSE_80	MAAPE_80
**Wheat_2**	Y	C	0.929	0.033	2.221	0.054
**Wheat_2**	Y	A	1.802	0.069	1.217	0.023

Dataset 8 Wheat_3

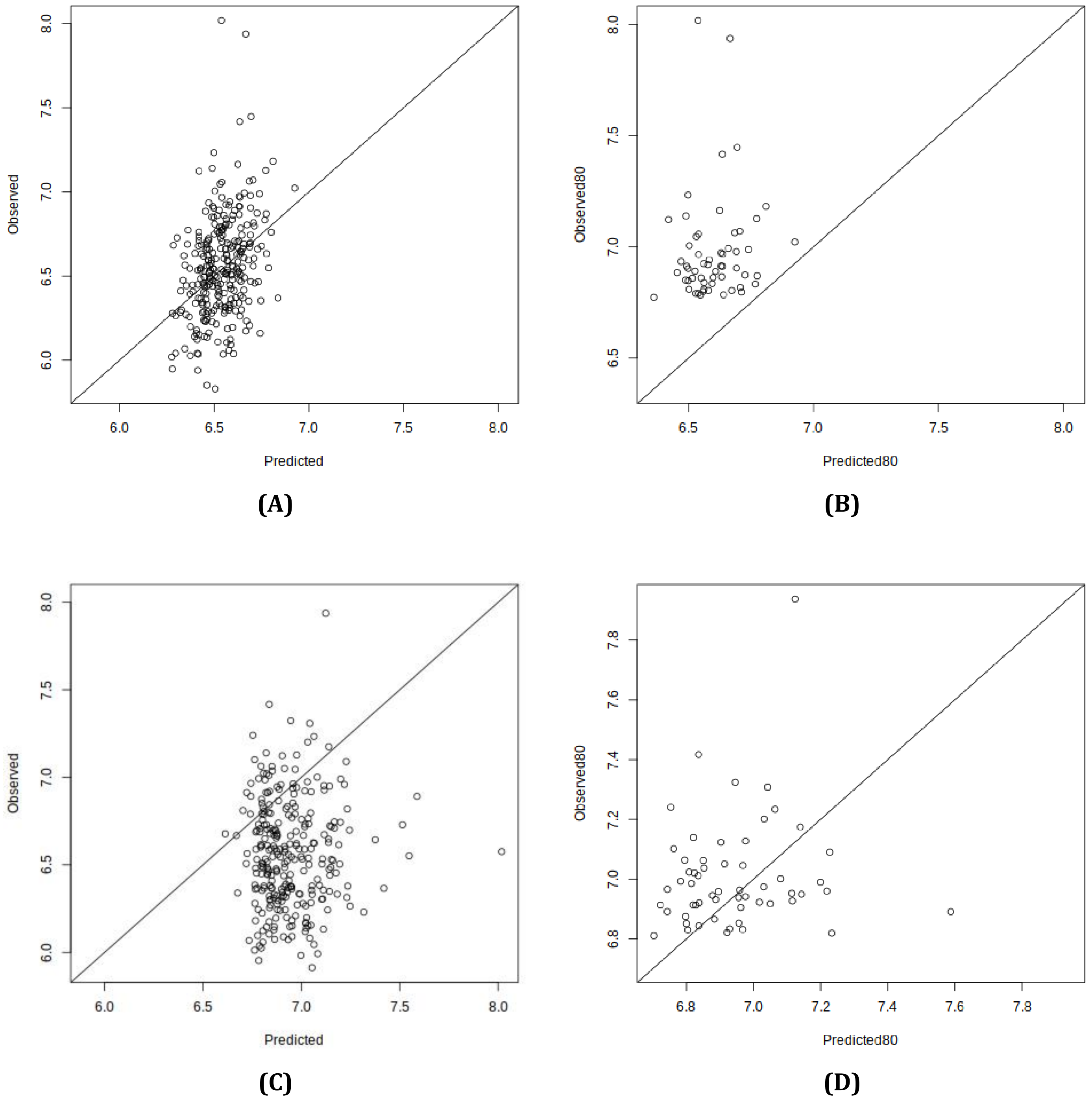

Figure S19. Prediction performance results for Dataset 8 **Wheat_3** in the trait **Y**, using the Conventional and Augmented methods, in terms of (**A**) the plots generated for the total testing using the Conventional method, (**B**) the plots generated for the top 20% of testing using the Conventional method, (**C**) the plots generated for the total testing using the Augmented method, (**D**) the plots generated for the top 20% of testing using the Augmented method.

Table S8. Prediction Accuracy Results for Dataset 8 **Wheat_3** using the C and A Methods, with Metrics MAAPE and NRMSE for the total testing, and MAAPE and NRMSE for the top 20% testing.

Dataset	Trait	Method	NRMSE	MAAPE	NRMSE_80	MAAPE_80
**Wheat_3**	Y	C	0.934	0.031	2.456	0.049
**Wheat_3**	Y	A	1.769	0.069	1.227	0.023

Dataset 9 Wheat_4

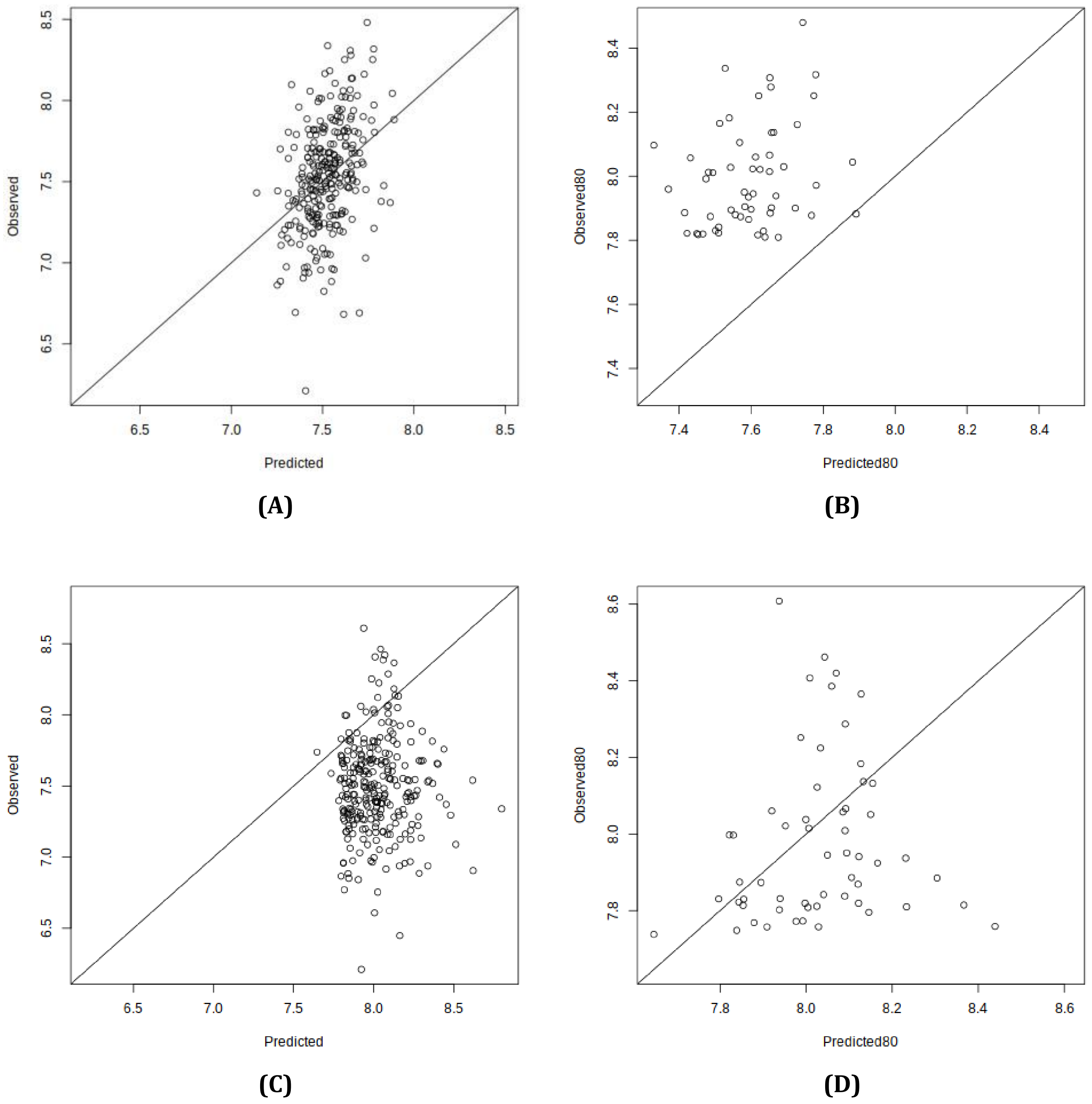

Figure 20. Prediction performance results for Dataset 9 **Wheat_4** in the trait **Y**, using the Conventional and Augmented methods, in terms of (**A**) the plots generated for the total testing using the Conventional method, (**B**) the plots generated for the top 20% of testing using the Conventional method, (**C**) the plots generated for the total testing using the Augmented method, (**D**) the plots generated for the top 20% of testing using the Augmented method.

Table S9. Prediction Accuracy Results for Dataset 9 **Wheat_4** using the C and A Methods, with Metrics MAAPE and NRMSE for the total testing, and MAAPE and NRMSE for the top 80% testing.

Dataset	Trait	Method	NRMSE	MAAPE	NRMSE_80	MAAPE_80
**Wheat_4**	Y	C	0.931	0.031	2.393	0.048
**Wheat_4**	Y	A	1.839	0.071	1.206	0.022

Dataset 10 Wheat _5

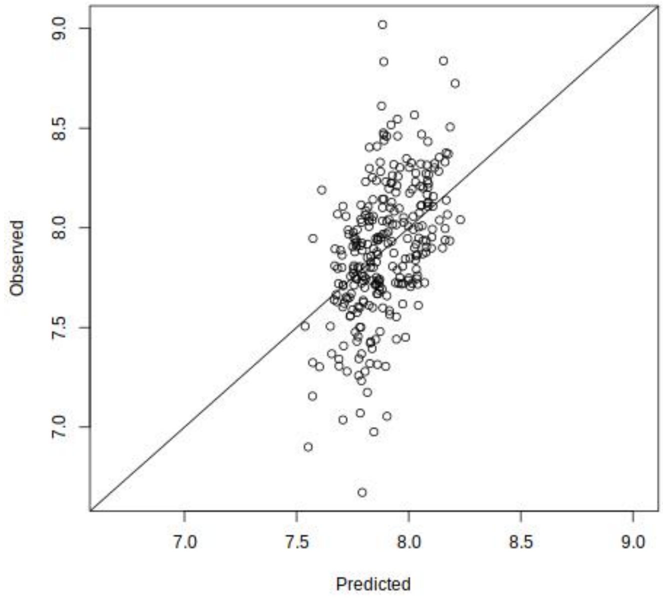

(A)

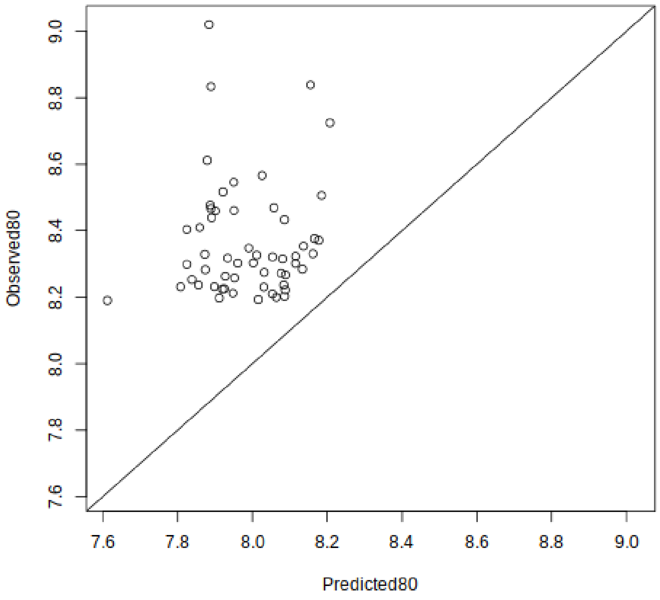

(B)

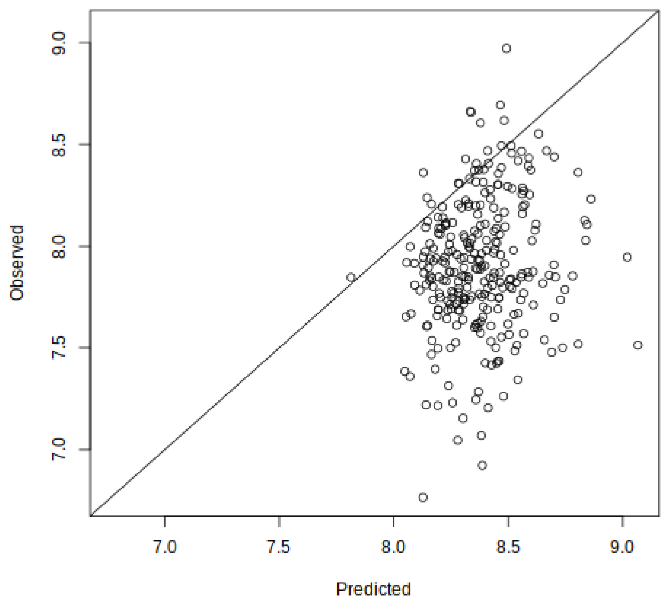

(C)

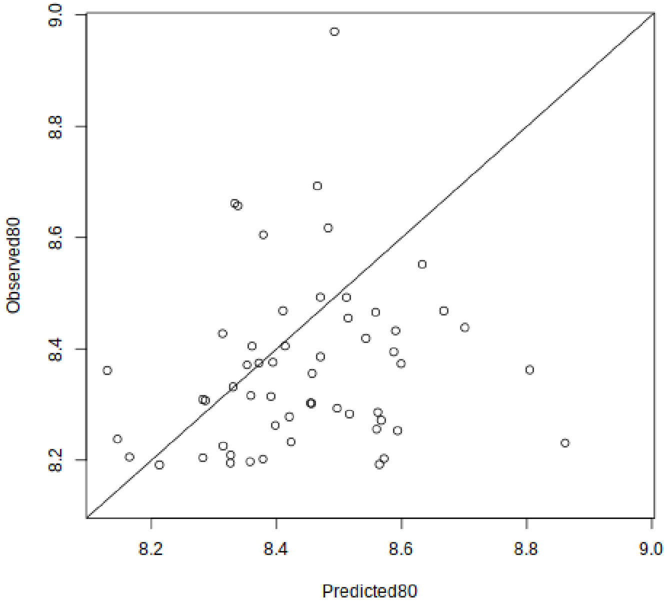

(D)

Figure S21. Prediction performance results for Dataset 10 **Wheat_5** in the trait **Y**, using the Conventional and Augmented methods, in terms of (**A**) the plots generated for the total testing using the Conventional method, (**B**) the plots generated for the top 20% of testing using the Conventional method, (**C**) the plots

generated for the total testing using the Augmented method, (**D**) the plots generated for the top 20% of testing using the Augmented method.

Table S10. Prediction Accuracy Results for Dataset **Wheat_5** using the C and A Methods, with Metrics MAAPE and NRMSE for the total testing, and MAAPE and NRMSE for the top 20% testing.

Dataset	Trait	Method	NRMSE	MAAPE	NRMSE_80	MAAPE_80
**Wheat_5**	Y	C	0.897	0.029	2.418	0.045
**Wheat_5**	Y	A	1.763	0.064	1.272	0.021

Dataset 11 Wheat_6

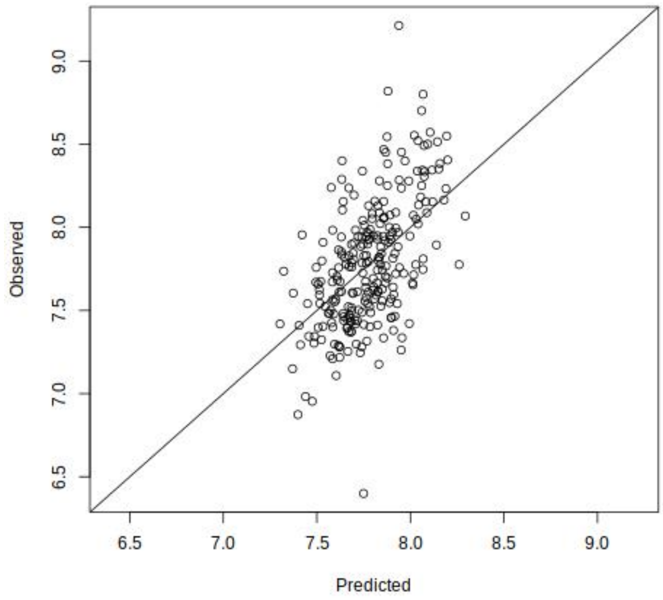

(A)

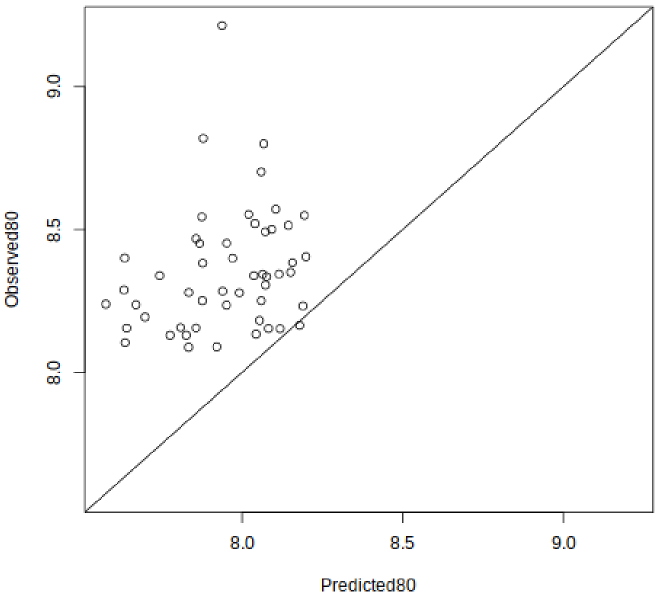

(B)

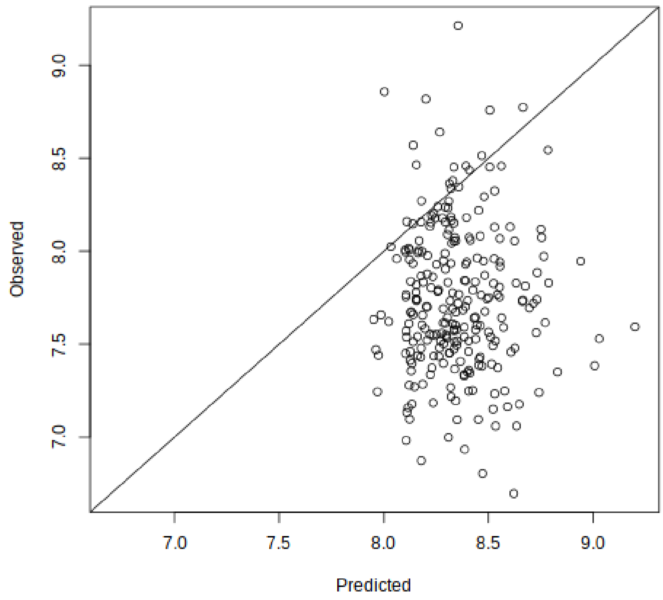

(C)

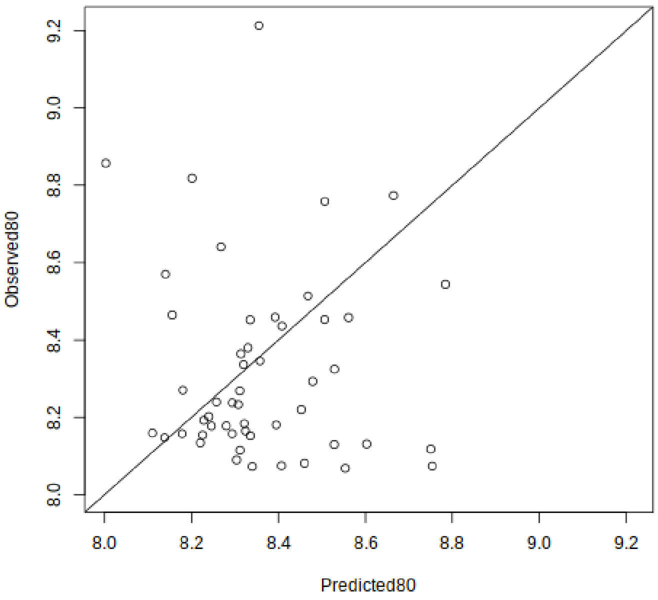

(D)

Figure S22. Prediction performance results for Dataset 11 **Wheat_6** in the trait **Y**, using the Conventional and Augmented methods, in terms of (**A**) the plots generated for the total testing using the Conventional method, (**B**) the plots generated for the top 20% of testing using the Conventional method, (**C**) the plots

generated for the total testing using the Augmented method, (**D**) the plots generated for the top 20% of testing using the Augmented method.

Table S11. Prediction Accuracy Results for Dataset 11 **Wheat_6** using the C and A Methods, with Metrics MAAPE and NRMSE for the total testing, and MAAPE and NRMSE for the top 20% testing.

Dataset	Trait	Method	NRMSE	MAAPE	NRMSE_80	MAAPE_80
**Wheat_6**	Y	C	0.844	0.033	2.140	0.046
**Wheat_6**	Y	A	1.858	0.080	1.293	0.026

Dataset 12 Indica

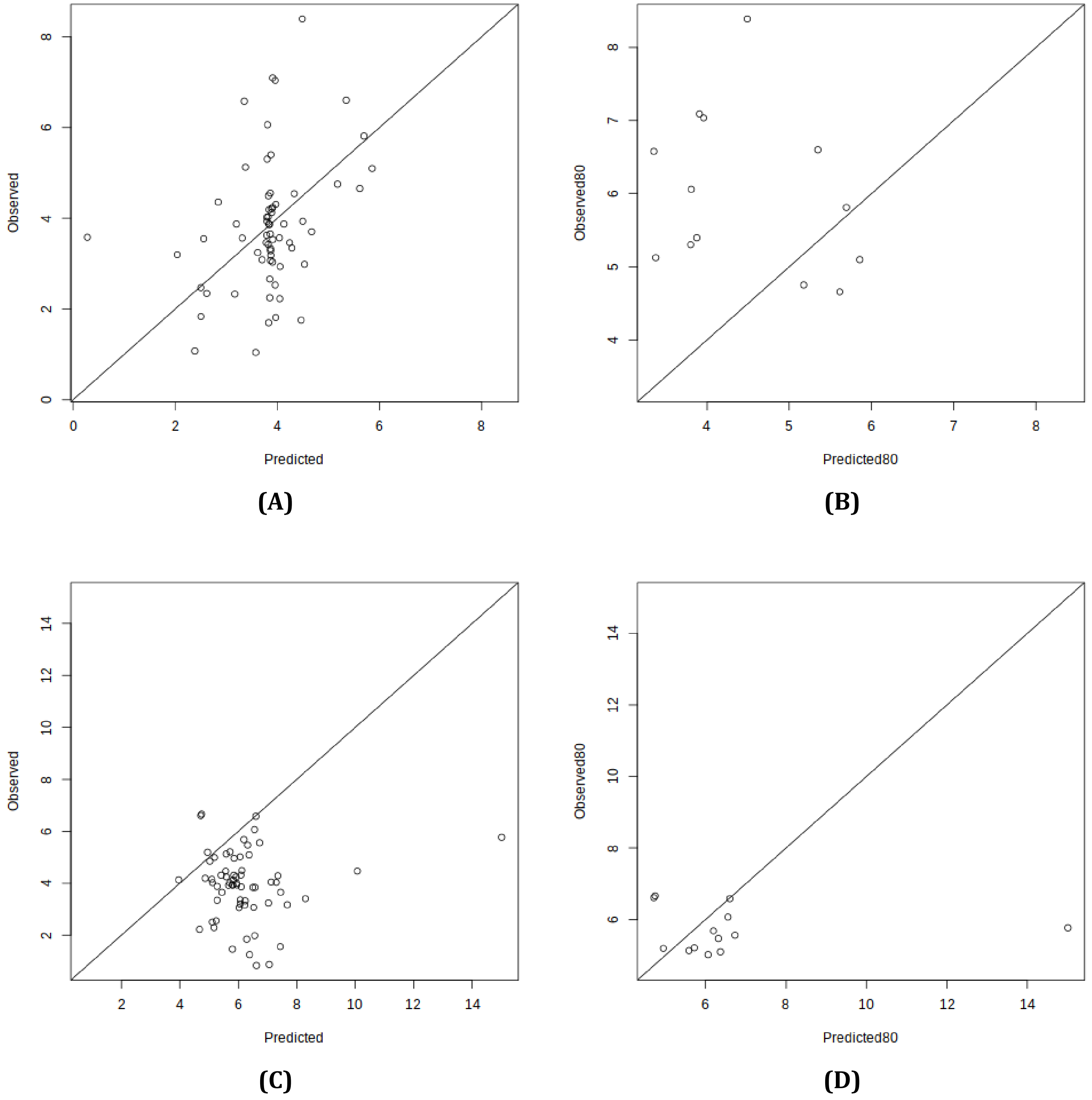

Figure S23. Prediction performance results for Dataset 12 **Indica** in the trait **GC**, using the Conventional and Augmented methods, in terms of (**A**) the plots generated for the total testing using the Conventional method, (**B**) the plots generated for the top 20% of testing using the Conventional method, (**C**) the plots generated for the total testing using the Augmented method, (**D**) the plots generated for the top 20% of testing using the Augmented method.

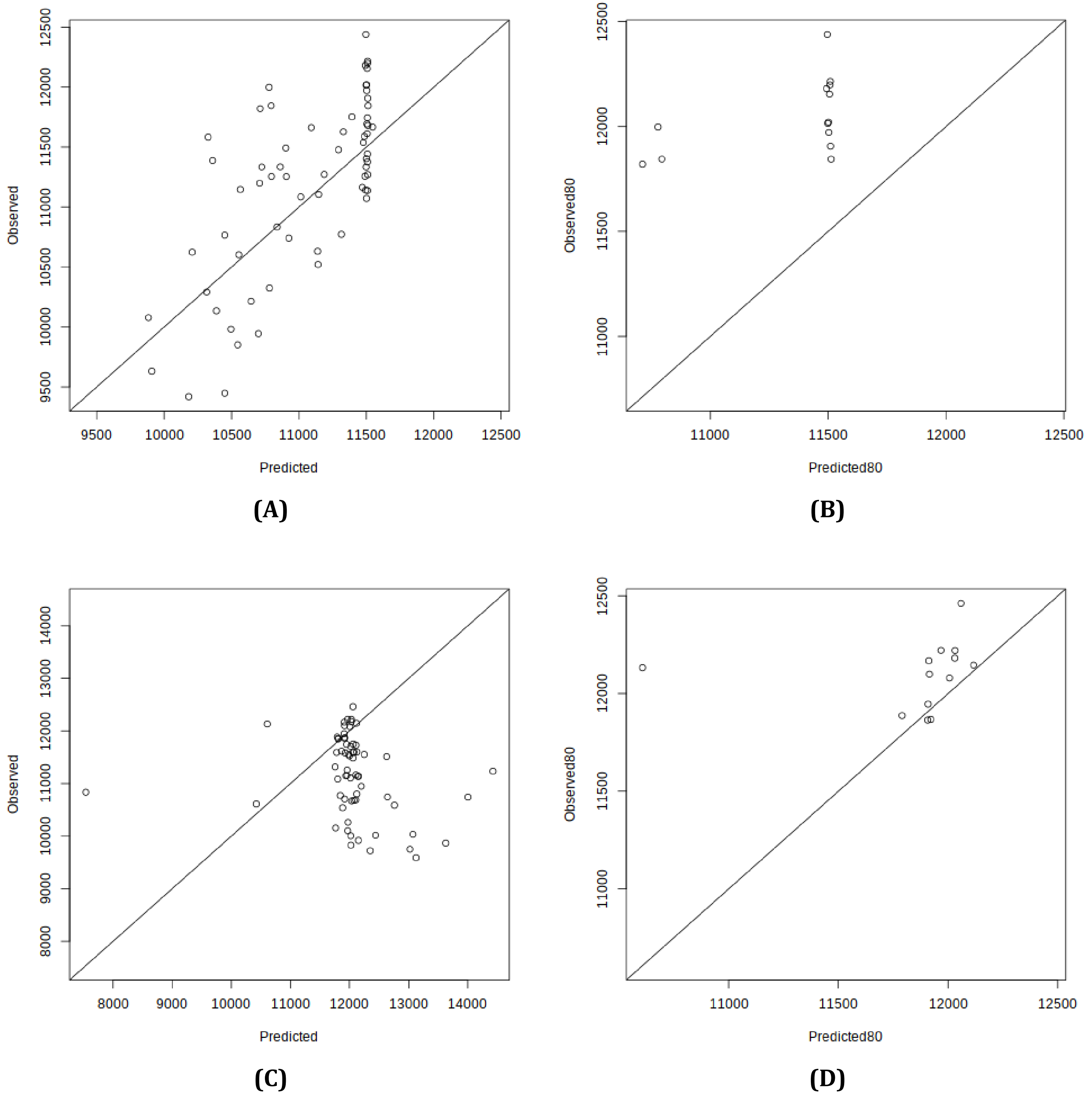

Figure S24. Prediction accuracy performance results for Dataset 12 **Indica** in the trait **GY**, using the Conventional and Augmented methods, in terms of (**A**) the plots generated for the total testing using the Conventional method, (**B**) the plots generated for the top 20% of testing using the Conventional method, (**C**) plots generated for the total testing using the Augmented method, (**D**) the plots generated for the top 20% of testing using the Augmented method.

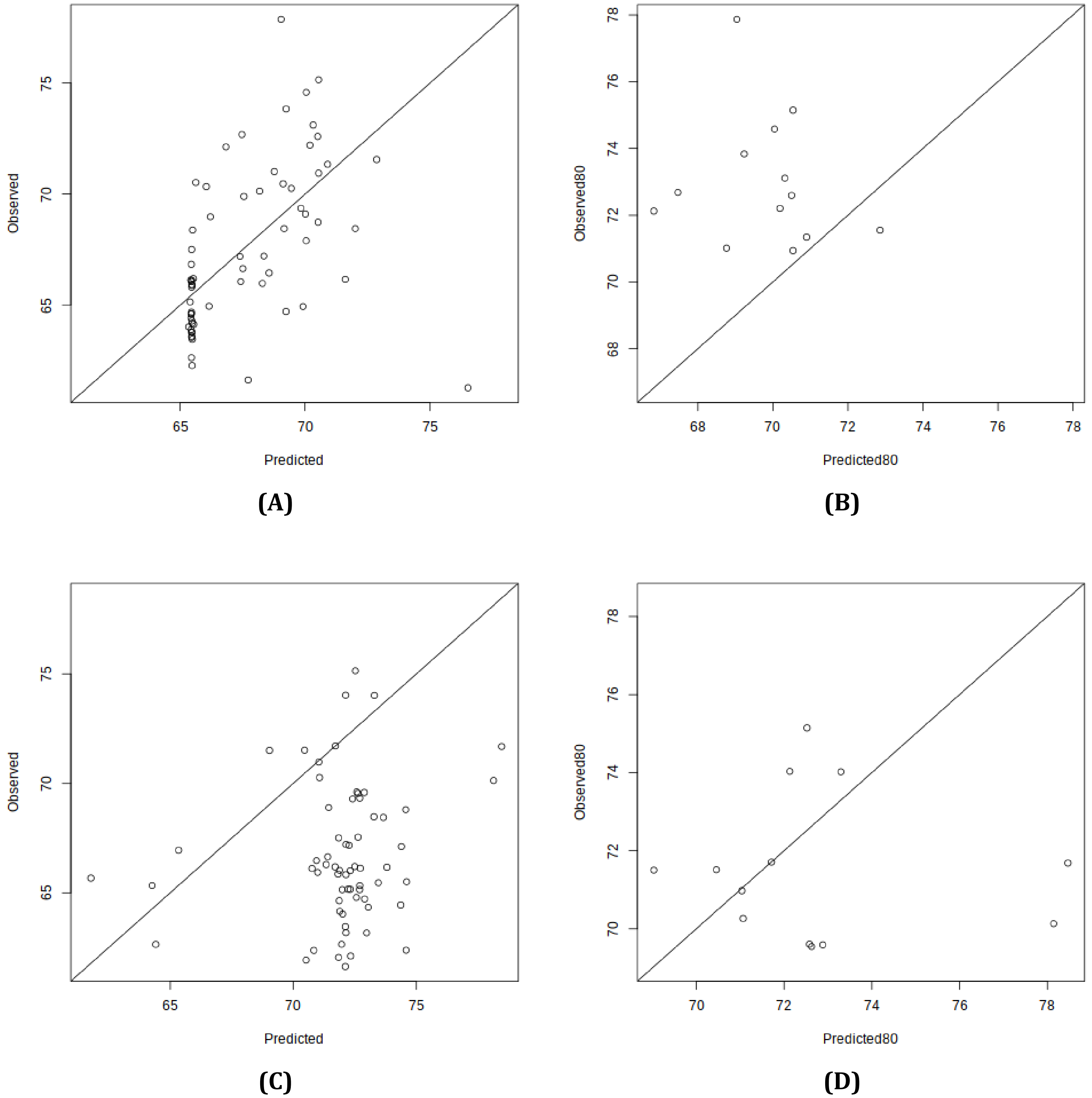

Figure S25. Prediction accuracy performance results for Dataset 12 **Indica** in the trait **PH**, using the Conventional and Augmented methods, in terms of (**A**) the plots generated for the total testing using the Conventional method, (**B**) plots generated for the top 20% of testing using the Conventional method, (**C**) plots generated for the total testing using the Augmented method, (**D**) the plots generated for the top 20% of testing using the Augmented method.

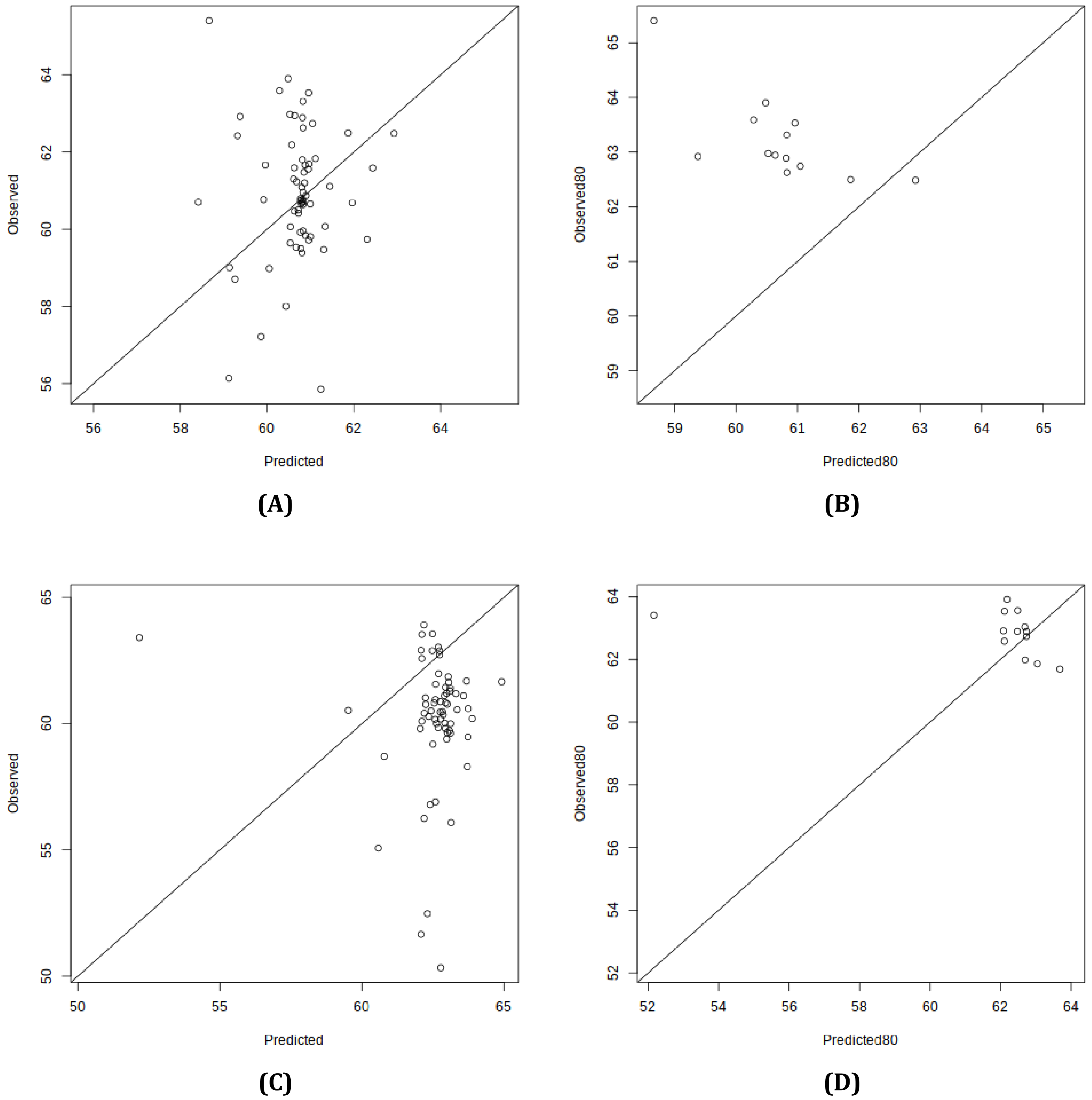

Figure 26. Prediction accuracy performance results for Dataset 12 **Indica** in the trait **PHR**, using the Conventional and Augmented methods, in terms of (**A**) the plots generated for the total testing using the Conventional method, (**B**) the plots generated for the top 20% of testing using the Conventional method, (**C**) the plots generated for the total testing using the Augmented method, (**D**) the plots generated for the top 20% of testing using the Augmented method.

Table S12. Prediction Accuracy Results for Dataset 12 **Indica** using the C and A Methods, with Metrics MAAPE and NRMSE for the total testing, and MAAPE and NRMSE for the top 20% testing.

Dataset	**Trait**	**Method**	**NRMSE**	**MAAPE**	**NRMSE_80**	**MAAPE_80**
Indica	GC	C	0.951	0.310	2.083	0.317
Indica	GC	A	1.869	0.571	1.722	0.201
Indica	GY	C	0.744	0.039	3.515	0.052
Indica	GY	A	1.817	0.103	1.817	0.023
Indica	PH	C	0.845	0.030	2.351	0.046
Indica	PH	A	1.831	0.083	1.506	0.027
Indica	PHR	C	0.943	0.024	3.509	0.033
Indica	PHR	A	1.557	0.047	2.300	0.017
Indica	AT	C	0.844	0.031	3.125	0.043
Indica	AT	A	1.735	0.078	1.874	0.022

Dataset 13 Japonica

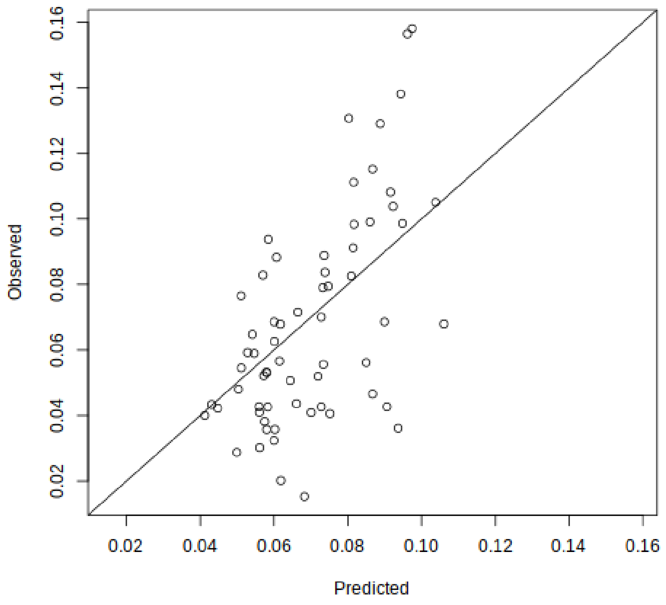

(A)

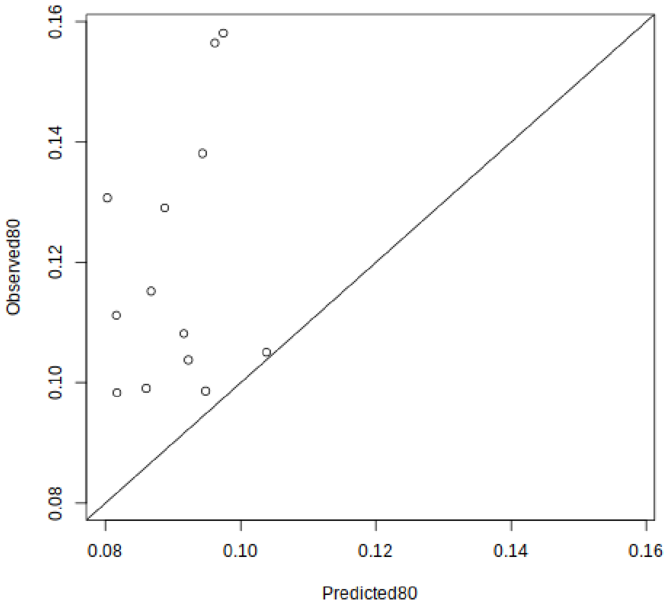

(B)

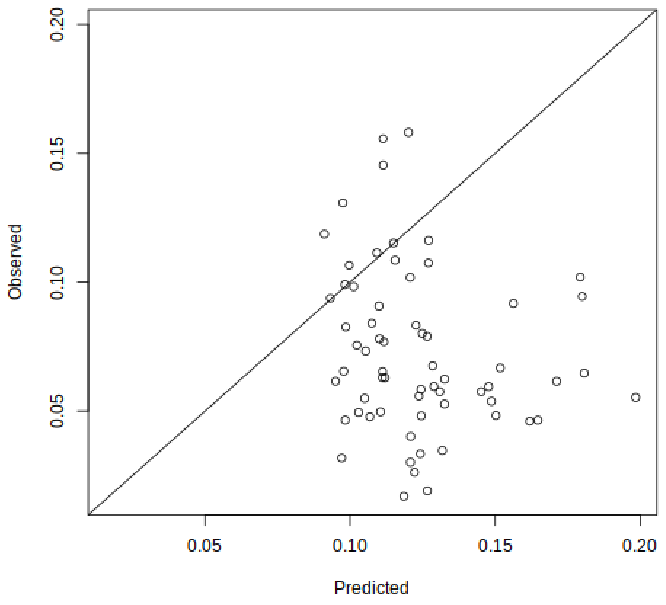

(C)

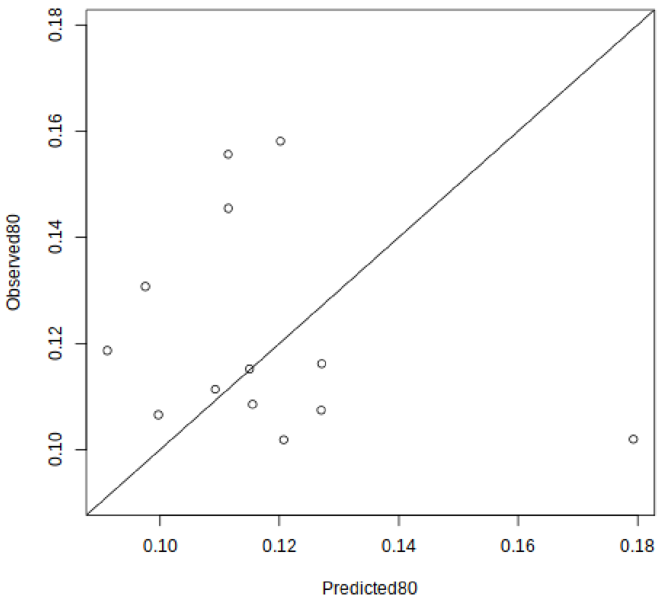

(D)

Figure S27. Prediction performance results for Dataset 13 **Japonica** in the trait **GC**, using the Conventional and Augmented methods, in terms of (**A**) the plots generated for the total testing using the Conventional method, (**B**) plots generated for the top 20% of testing using the Conventional method, (**C**) the plots generated for the total testing using the Augmented method, (**D**) the plots generated for the top 20% of testing using the Augmented method.

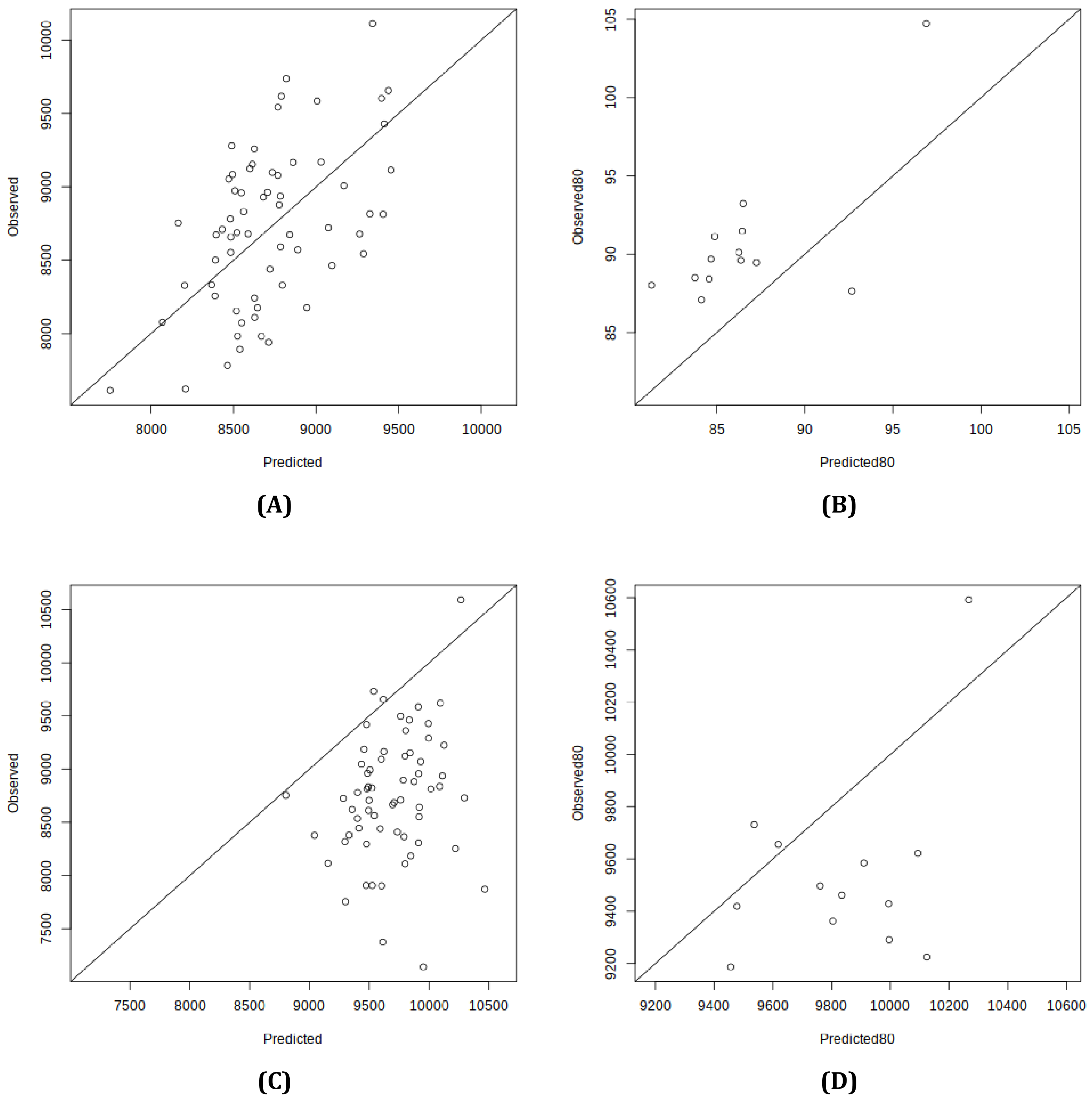

Figure S28. Prediction accuracy performance results for Dataset 13 **Japonica** in the trait **GY**, using the Conventional and Augmented methods, in terms of (**A**) the plots generated for the total testing using the Conventional method, (**B**) the plots generated for the top 20% of testing using the Conventional method, (**C**) the plots generated for the total testing using the Augmented method, (**D**) the plots generated for the top 20% of testing using the Augmented method.

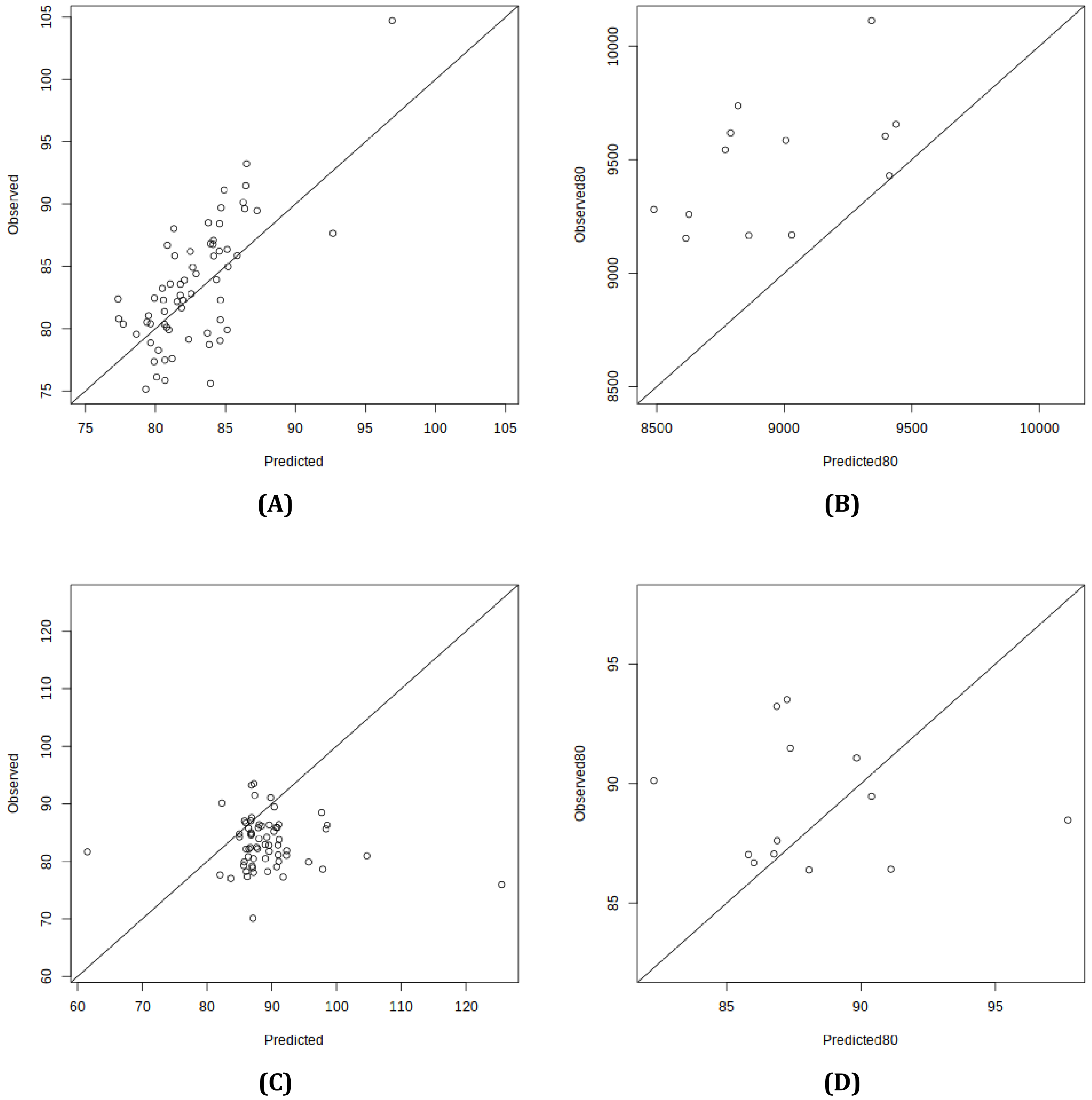

Figure S29. Prediction accuracy performance results for Dataset 13 **Japonica** in the trait **PH**, using the Conventional and Augmented methods, in terms of (**A**) the plots generated for the total testing using the Conventional method, (**B**) the plots generated for the top 20% of testing using the Conventional method, (**C**) the plots generated for the total testing using the Augmented method, (**D**) plots generated for the top 20% of testing using the Augmented method.

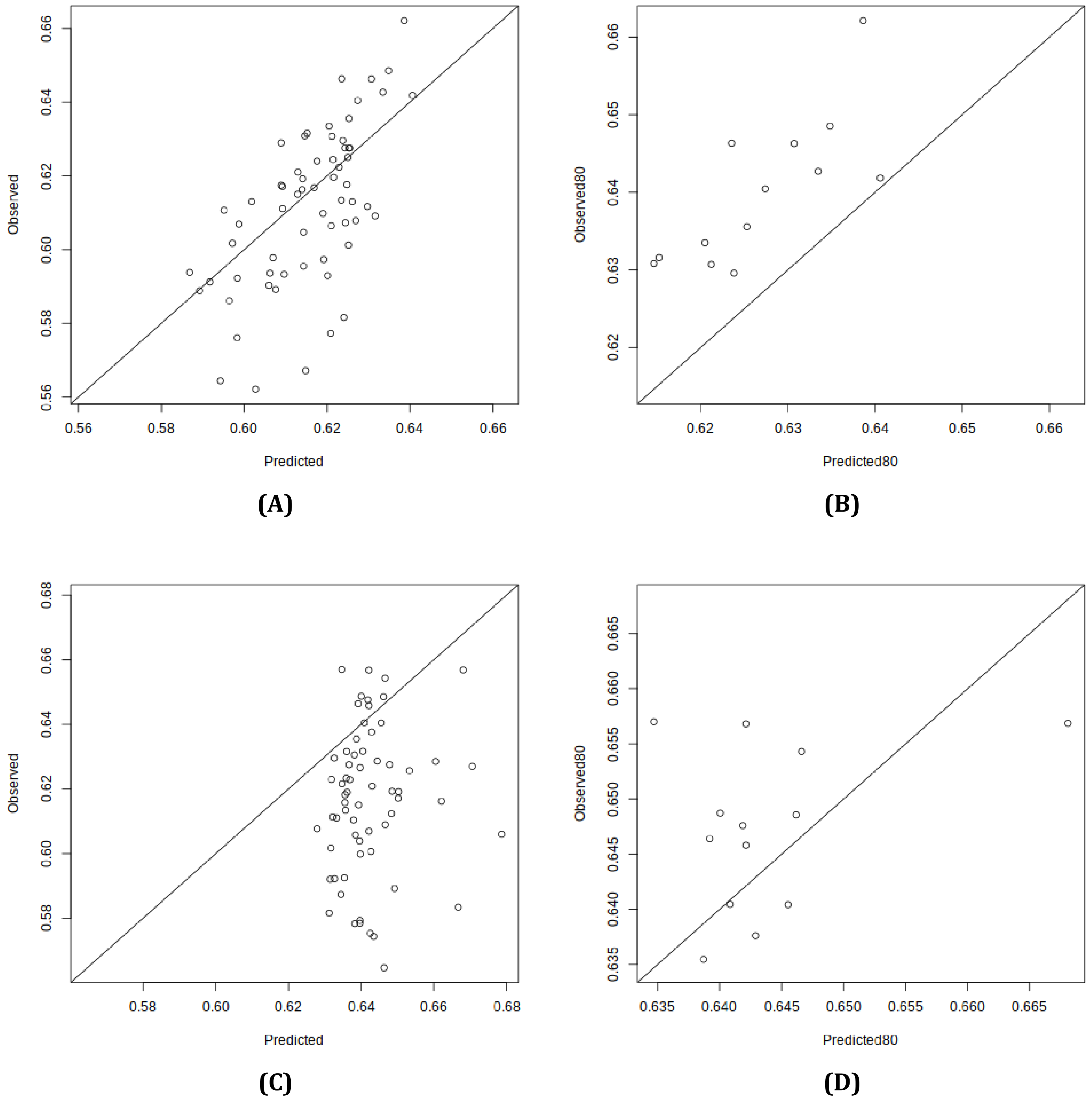

Figure S30. Prediction performance results for Dataset 13 **Japonica** in the trait **PHR**, using the Conventional and Augmented methods, in terms of (**A**) the plots generated for the total testing using the Conventional method, (**B**) the plots generated for the top 20% of testing using the Conventional method, (**C**) the plots generated for the total testing using the Augmented method, (**D**) the plots generated for the top 20% of testing using theAugmented method.

Table S13. Prediction Accuracy Results for Dataset 13 **Japonica** using the C and A Methods, with Metrics MAAPE and NRMSE for the total testing, and MAAPE and NRMSE for the top 20% testing.

Dataset	**Trait**	**Method**	**NRMSE**	**MAAPE**	**NRMSE_80**	**MAAPE_80**
Japonica	GC	C	0.840	0.320	1.909	0.251
Japonica	GC	A	1.915	0.644	1.537	0.195
Japonica	GY	C	0.845	0.0482	2.115	0.062
Japonica	GY	A	1.833	0.117	1.595	0.038
Japonica	PH	C	0.795	0.032	2.073	0.050
Japonica	PH	A	1.922	0.088	1.412	0.042
Japonica	PHR	C	0.860	0.024	3.361	0.031
Japonica	PHR	A	1.695	0.048	1.219	0.011
Japonica	AT	C	0.833	0.035	2.516	0.048
Japonica	AT	A	1.817	0.085	1.409	0.030

Data set 14 Groundnut

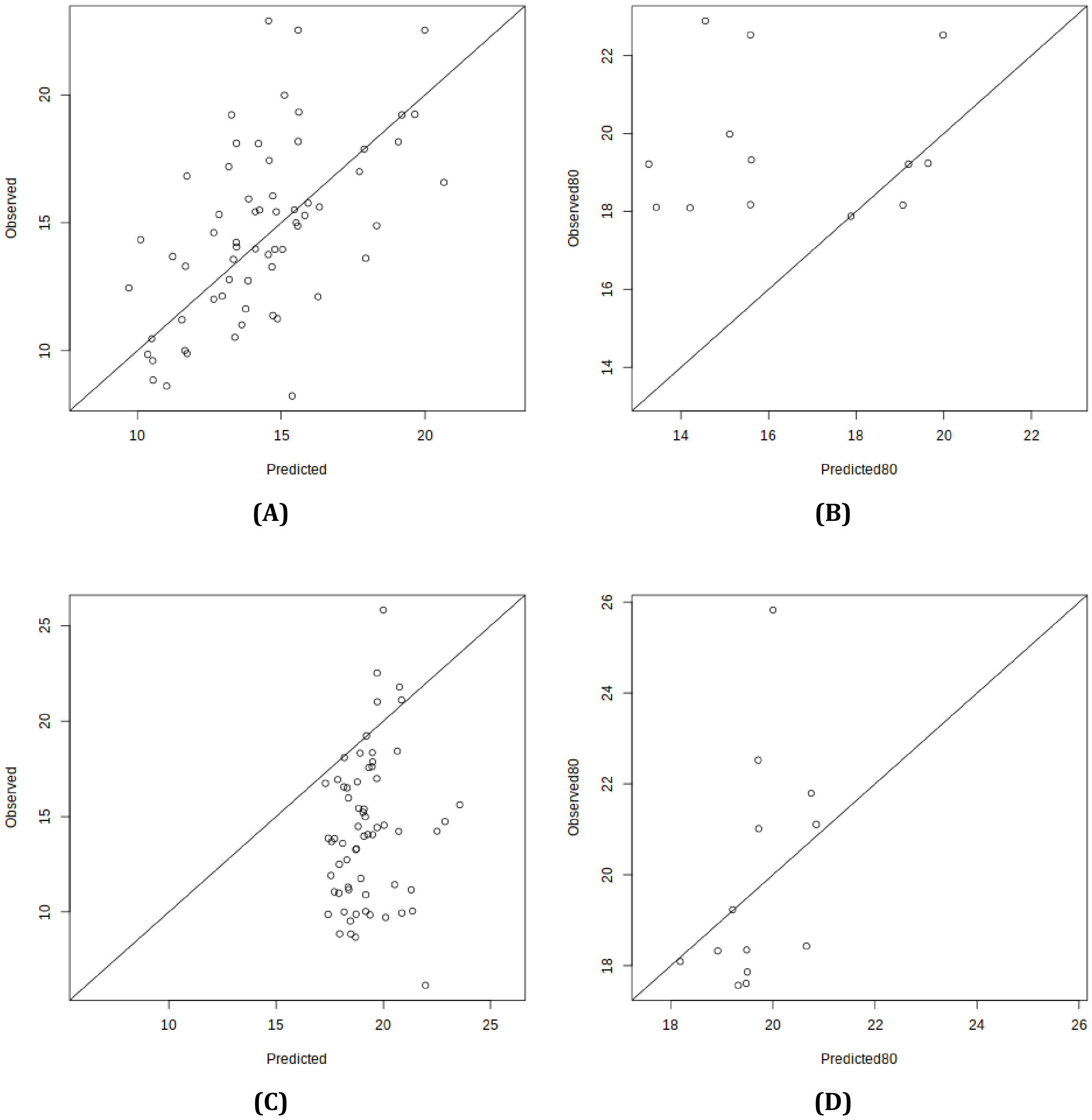

Figure S31. Prediction performance results for Dataset 14 **Groundnut** in the trait **NPP**, using the Conventional and Augmented methods, in terms of (**A**) the plots generated for the total testing using the Conventional method, (**B**) plots generated for the top 20% of testing using the Conventional method, (**C**) plots generated for the total testing using the Augmented method, (**D**) plots generated for the top 20% of testing using the Augmented method.

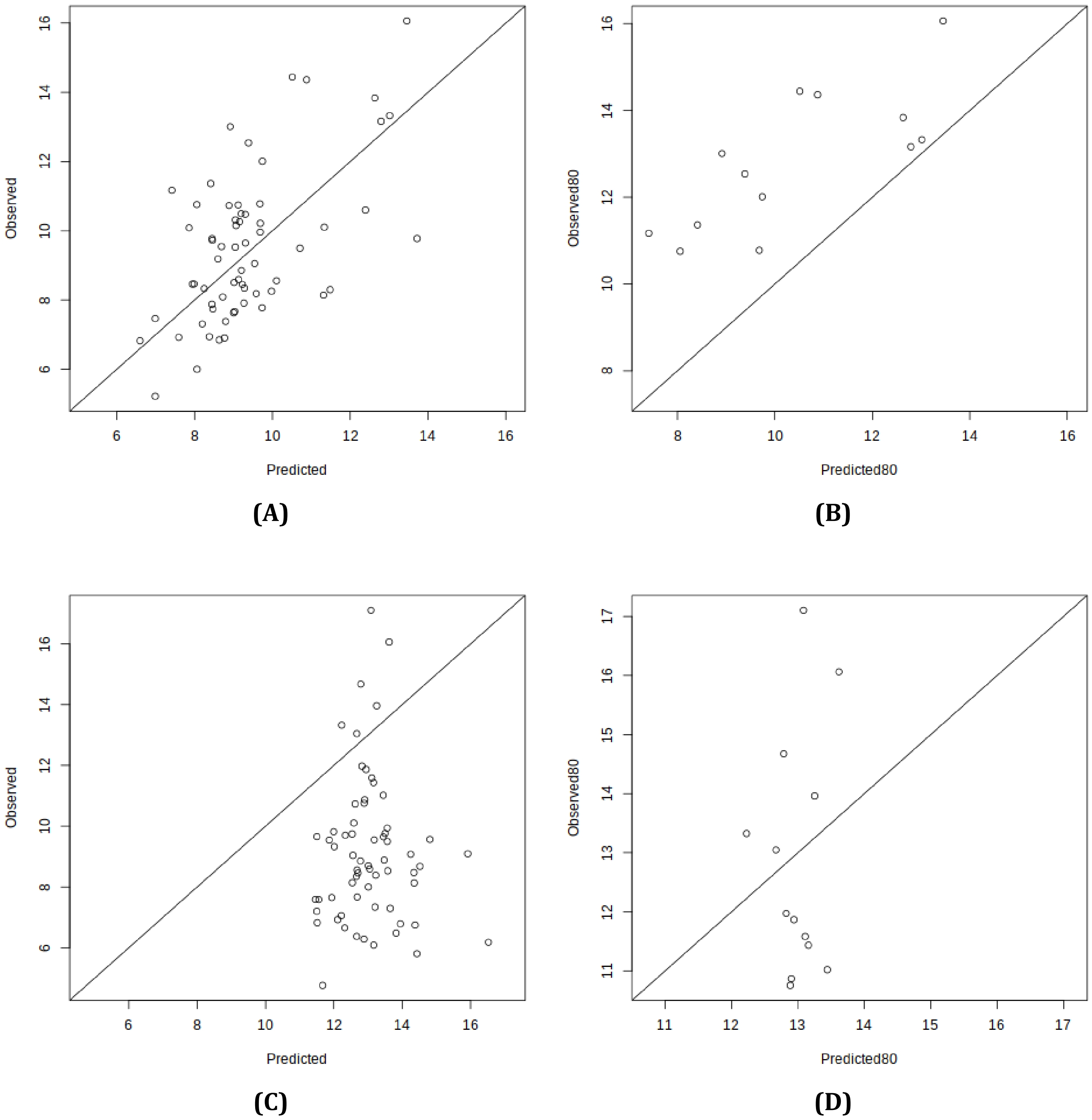

Figure S32. Prediction accuracy performance results for Dataset 14 **Groundnut** in the trait **PYPP**, using the Conventional and Augmented methods, in terms of (**A**) plots generated for the total testing using the Conventional method, (**B**) the plots generated for the top 20% of testing using the Conventional method, (**C**) plots generated for the total testing using the Augmented method, (**D**) plots generated for the top 20% of testing using the Augmented method.

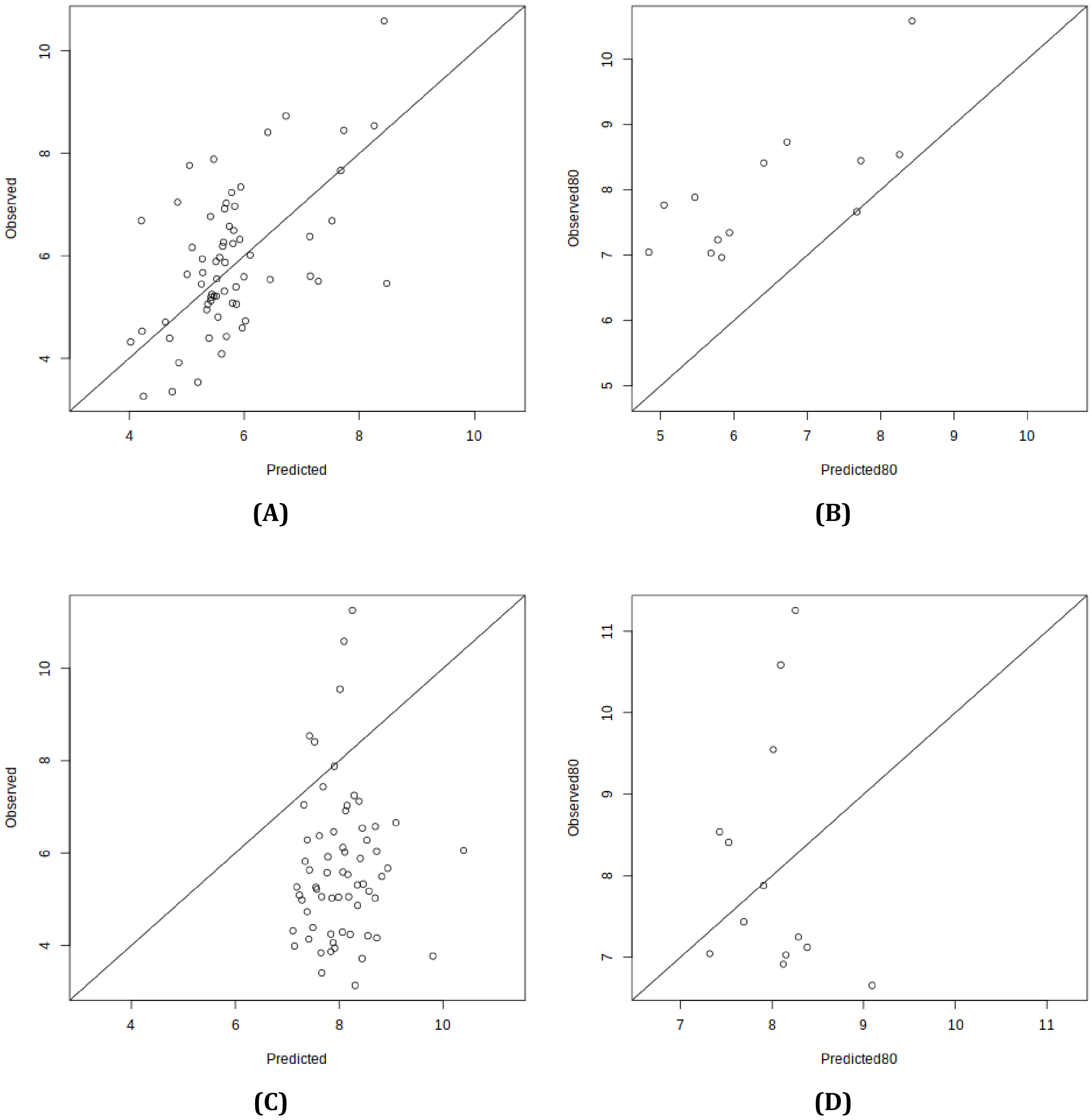

Figure S33. Prediction accuracy performance results for Dataset 14 **Groundnut** in the trait **SYPP**, using the Conventional and Augmented methods, in terms of (**A**) the plots generated for the total testing using Conventional method, (**B**) the plots generated for the top 20% of testing using the Conventional method, (**C**) the plots generated for the total testing using theAugmented method, (**D**) the plots generated for the top 20% of testing using the Augmented method.

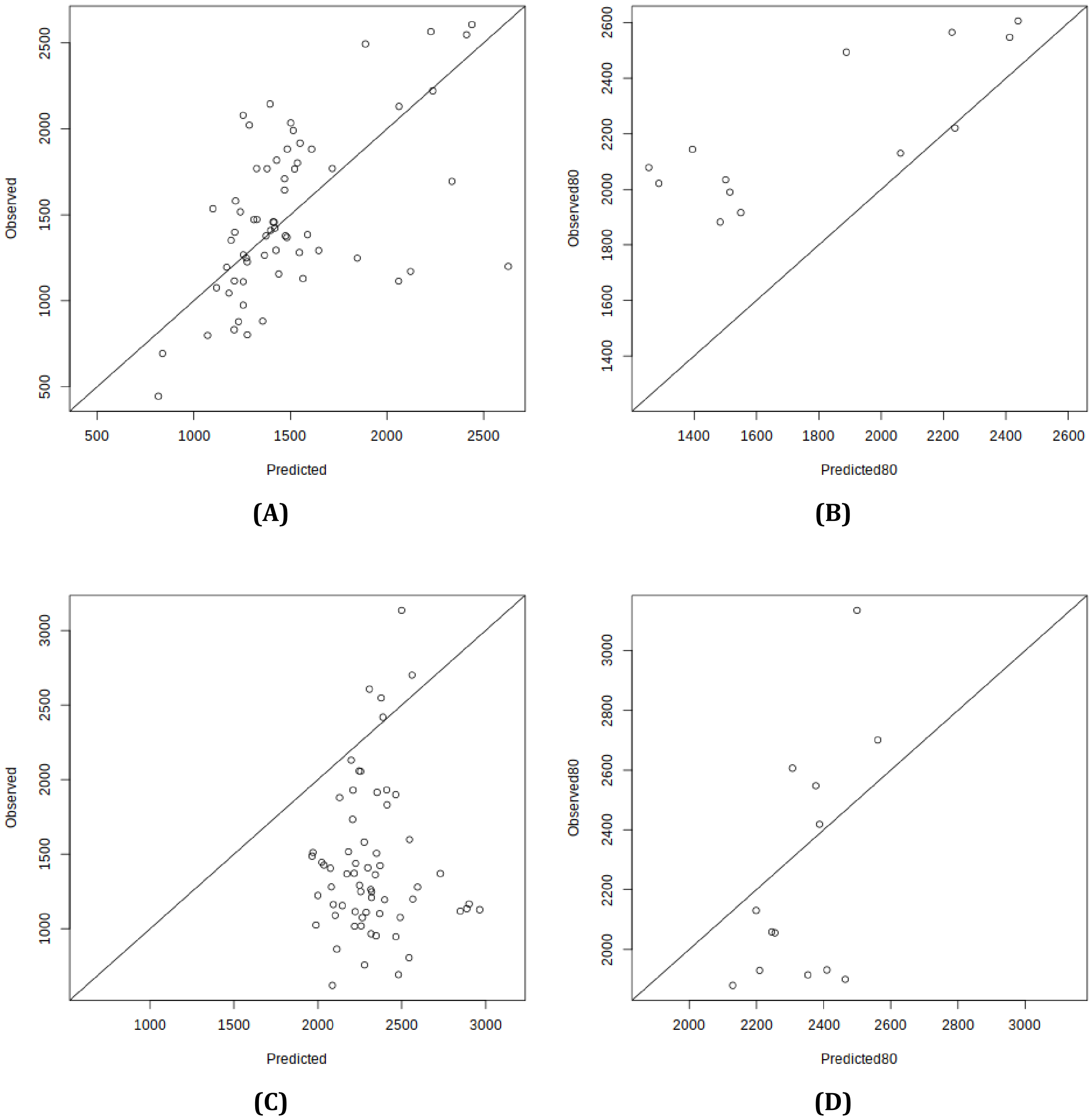

Figure S34. Prediction accuracy performance results for Dataset 14 **Groundnut** in the trait **YPH**, using the Conventional and Augmented methods, in terms of (**A**) the plots generated for the total testing using Conventional method, (**B**) the plots generated for the top 20% of testing using the Conventional method, (**C**) plots generated for the total testing using the Augmented method, (**D**) plots generated for the top 20% of testing using the Augmented method.

Table 14. Prediction Accuracy Results for Dataset **Groundnut** using the C and A Methods, with Metrics MAAPE and NRMSE for the total testing, and MAAPE and NRMSE for the top 20% testing.

Dataset	**Trait**	**Method**	**NRMSE**	**MAAPE**	**NRMSE_80**	**MAAPE_80**
Groundnut	NPP	C	0.738	0.156	1.897	0.155
Groundnut	NPP	A	1.831	0.406	1.290	0.104
Groundnut	PYPP	C	0.773	0.159	1.730	0.181
Groundnut	PYPP	A	1.994	0.439	1.274	0.113
Groundnut	SYPP	C	0.788	0.167	1.779	0.183
Groundnut	SYPP	A	1.975	0.449	1.363	0.121
Groundnut	YPH	C	0.745	0.209	1.876	0.208
Groundnut	YPH	A	2.057	0.592	1.236	0.135
Groundnut	AT	C	0.769	0.178	1.795	0.191
Groundnut	AT	A	2.009	0.493	1.291	0.123
